# Sequence features responsible for intron retention in human

**DOI:** 10.1186/1471-2164-8-59

**Published:** 2007-02-26

**Authors:** Noboru Jo Sakabe, Sandro José de Souza

**Affiliations:** 1 Ludwig Institute for Cancer Research, Sao Paulo Branch, Hospital Alemão Oswaldo Cruz, Rua João Julião, 245 – 1° andar, CEP 01323-903, São Paulo, SP, Brazil; 2 PhD. Program, Departamento de Bioquímica, Universidade de São Paulo, Av. Prof. Lineu Prestes, 748 – Bloco 03 superior, sala 351, CEP: 05508-900, Cidade Universitária, São Paulo, SP, Brazil

## Abstract

**Background:**

One of the least common types of alternative splicing is the complete retention of an intron in a mature transcript. Intron retention (IR) is believed to be the result of intron, rather than exon, definition associated with failure of the recognition of weak splice sites flanking short introns. Although studies on individual retained introns have been published, few systematic surveys of large amounts of data have been conducted on the mechanisms that lead to IR.

**Results:**

TTo understand how sequence features are associated with or control IR, and to produce a generalized model that could reveal previously unknown signals that regulate this type of alternative splicing, we partitioned intron retention events observed in human cDNAs into two groups based on the relative abundance of both isoforms and compared relevant features. We found that a higher frequency of IR in human is associated with individual introns that have weaker splice sites, genes with shorter intron lengths, higher expression levels and lower density of both a set of exon splicing silencers (ESSs) and the intronic splicing enhancer GGG. Both groups of retained introns presented events conserved in mouse, in which the retained introns were also short and presented weaker splice sites.

**Conclusion:**

Although our results confirmed that weaker splice sites are associated with IR, they showed that this feature alone cannot explain a non-negligible fraction of events. Our analysis suggests that cis-regulatory elements are likely to play a crucial role in regulating IR and also reveals previously unknown features that seem to influence its occurrence. These results highlight the importance of considering the interplay among these features in the regulation of the relative frequency of IR.

## Background

Most eukaryotic genes are composed by exons and introns, requiring pre-mRNA splicing. Although splicing occurs with incredible fidelity, a high rate of alternative joining of exons has been observed. Alternative splicing can be mainly of three types: *exon skipping*, whereby an exon may be included or not in the mature mRNA, *alternative use of splice sites*, resulting in longer or shorter exons and *intron retention (IR)*, whereby an intron sequence is maintained in the mature transcript or spliced out of it (reviewed in [1]).

Intron retention is unexpected since it affects mRNA transport to the cytoplasm [2] and can insert premature stop codons in the mature transcript that would then be degraded by non-sense mediated decay [3]. In fact, decreasing the levels of mRNAs of a given gene can be a function of alternative splicing [3]. This, however, is not the fate of all mRNAs with a retained intron. For example, an isoform of the mouse *tgif2 *gene that retains an intron in the coding region was shown to have biological activity [4]. Furthermore, we have previously shown that introns retained in the human transcriptome bear signals of being biologically functional as GC content and codon usage similar to those of coding exons and the capacity of encoding protein domains [5].

In respect to the mechanistic nature of IR, the most intuitive explanation is that these introns are flanked by weak splice sites that occasionally are not properly recognized. Stamm and coworkers [6] observed weaker conservation of splice sites flanking retained introns. Studies with human genes have shown that strengthening sub-optimal splice sites flanking retained introns caused an increase in their removal levels [7] or completely abolished retention [8]. On the other hand, other studies showed that weakening optimal splice sites leads to intron retention (chicken troponin I gene [9], human α-globin 2 [10], *Drosophila melanogaster*'s *zeste *[11], *S. pombe*'s *cdc2 *gene [12]).

Interestingly, all the introns involved in these studies were short (< 274 nt). This may have a mechanistic reason according to current models of splice site recognition. In the exon definition model [13], splice sites are recognized throughout the exons, which in vertebrates are usually shorter than introns. In this case, the non-recognition of a pair of weak splice sites would lead to the skipping of an exon, rather than to IR. However, in organisms whose exons are normally longer than introns (*S. pombe*,*C. elegans *and *Drosophila*, for instance), splice site pairs seem to be recognized throughout introns, the intron definition model [13]. The prevalence of one mechanism over the other seems to be based on the recognition of the shortest unit, either an intron or an exon over which a protein "bridge" connects two exon borders [11]. Therefore, even in vertebrates, short introns could be the recognition unit for splicing and in this case IR is expected if splice sites are not properly recognized [11].

In addition to splice site variation, some studies showed that the expression of the intron retaining and intron spliced isoforms varies along tissues [5,14,15] indicating that cellular factors are also involved in the control of intron retention. In fact, splice site recognition depends on several factors as for example regulatory elements located in the mRNA sequence and on the concentration of protein factors (for review, see [1]). It has been shown that the presence of specific *cis*-regulatory elements can indeed counterbalance retention of individual introns [8,16].

Although the studies mentioned above provide an experimental basis for IR on specific genes, a generalized model based on a large quantity of data is still missing. This model could reveal how different factors contribute to the frequency of IR. We sought to identify potential sequence features that may be associated with this type of alternative splicing by examining the contrasting features of two sets of retained introns found in human cDNAs. One set corresponded to those cases in which the intron retaining isoform was the minor splice form and the other in which it was the major form. We found that a higher relative frequency of IR is associated with weaker splice sites, genes with overall short intron lengths, higher expression levels and particular densities of regulatory elements.

## Results

IR events were identified on human cDNA sequences aligned to the genome. An event was considered to be any occurrence of at least one cDNA defining an intron through the existence of its flanking exons plus at least one other spliced cDNA containing at least parts (> 1 nt) of the flanking exons and the intron (see Methods).

In order to understand how splice site strength controls IR, we analyzed retained introns partitioned into two categories, according to their relative isoform frequencies (RIF = number of cDNAs with retained intron/number of cDNAs defining the intron). The low-RIF group contained IR events where the intron retaining cDNA was the minor form (RIF < 1, which corresponds to < 50 % of the cDNAs with IR) and the high-RIF group contained those events where it was the major form (RIF > 1, > 50% of IR). As we wanted to contrast features of the groups of IR events with different relative frequencies of IR, events with an equal number of intron retaining and intron defining evidences (3% of all filtered cases) were not considered.

The initial set of events (6370 low and 1838 high-RIF) was filtered to produce a higher quality data set. Only those events where both the intron retaining and intron defining forms were confirmed by at least two cDNAs each, presenting no alternative borders (see Methods) and retaining introns longer than 50 nt were accepted. The filtered data set was composed by 1516 low and 296 high-RIF IR events.

A few retained introns flanked by non-canonical splice sites were found in the filtered data set (1 in the low and 46 in the high-RIF group). As they were not G(T/C)..AG introns and in several cases different non-canonical splice sites could be obtained by adjustments in the alignment, they were excluded from the filtered data set (also see Additional file 1: Comment on retained introns with non-canonical splice sites). Information about the final data sets is presented in Table 1.

**Table 1:** Partitioning of intron retention events in the human transcriptome according to their relative isoform frequencies (RIF).

RIF group	events	number of cDNA clusters*	number of clusters w/> 1 event of the same RIF**	number of events/cluster
low (< 50% retaining forms)	1515	1114	282	1.4
high (> 50% retaining forms)	250	244	6	1.0

* 53 clusters presented both low and high-RIF events (≥ 2 different retained introns in the same gene)

** ≥ 2 distinct retained introns in the same gene, see Discussion

### Splice site strength as a cause of intron retention

**Figure 1:**
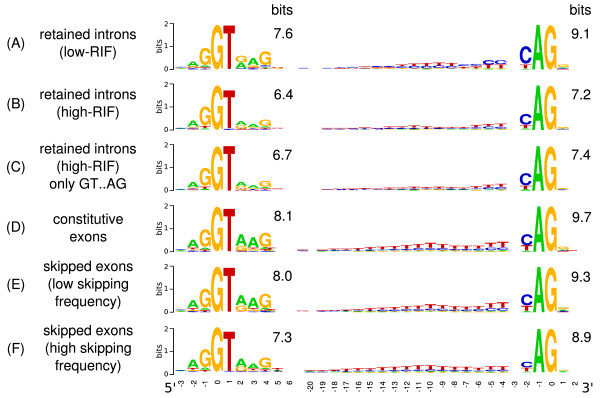
**Sequence logos for 5' and 3' splice sites of retained introns, constitutive and skipped exons**. The logos show splice sites flanking: (A) Low-RIF retained introns (1515 splice site pairs), (B) High-RIF retained-introns (250 pairs), (C) Only high-RIF retained introns flanked by GT..AG (240 pairs), (D) Constitutive exons (those that did not present any evidence of involvement in any type of alternative splicing, 90185 pairs), (E) Skipped exons with RIF < 1 (31414 pairs), (F) Skipped exons with RIF > 1 (9567 pairs). The RIF for exon skipping was calculated analogously for intron retention (RIF = number of cDNAs skipping the exon/cDNAs with the exon).

For each RIF group, 5' and 3' splice sites had their information contents calculated (Figure 1). The sequence logos show that splice sites of the low-RIF group (Figure 1A) resemble those that flank constitutive exons (Figure 1D) but the polypyrimidine tract is weaker when compared to constitutive exons. In the high-RIF group, splice sites are less conserved and present an even weaker polypyrimidine tract, indicating that the high frequency of IR is due to weaker splice sites (Figures 1B and 1C). Stamm and coworkers [6] also observed that retained introns present a higher deviation of the polypyrimidine tract in comparison to other types of alternative splicing. Also, it is interesting to note that short *Drosophila *introns do not present a strong polypyrimidine tract [17] and that its absence favors the intron definition model [11].

**Figure 2:**
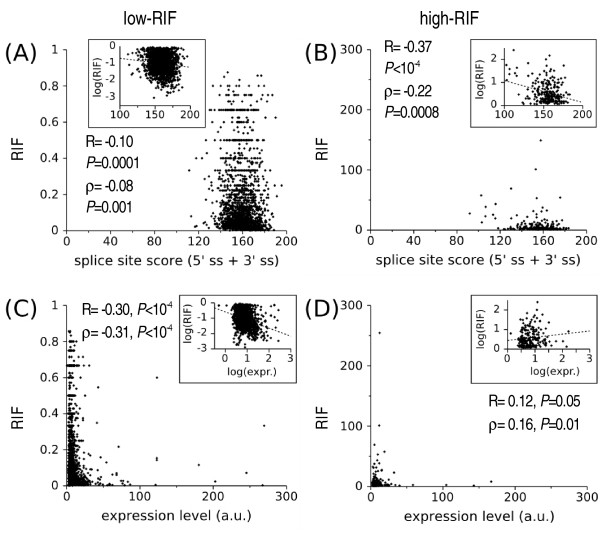
**Association of splice site strength, expression level and RIF**. Panels (A) and (B): Frequency of retention as a function of Shapiro and Senapathy scores (donor + acceptor). (C) and (D): Frequency of retention as a function of expression levels (1306 low-RIF and 203 high-RIF events for which there was SAGE data). Average expressions per gene were used. The insets show the log transformed data and the lines are the least squares regression fits. ρ = Spearman rank order and R=Pearson linear correlation coefficients (on log data). Note that the linear regression fittings should be taken only as an indicator of the tendency, rather than formal data fitting.

Reinforcing the idea that weak splice sites are a cause of IR, there is a weak negative correlation between splice site strength and the frequency of retention as measured using the Shapiro and Senapathy scoring scheme (S&S score [18]) and the RIF within each group (low-RIF: ρ = -0.08, *P *= 0.001, high-RIF: ρ = -0.22, *P *= 0.0008, Figure 2, panels A and B). The low correlation coefficients found suggest that the RIF is influenced by several other factors besides the splice site strength. Also, the RIF as calculated here is only a rough estimation since it does not take into account tissue variability, library normalization and possible cloning bias.

Another way to view the dependence of RIF on splice site strength is to compare the frequencies of splice sites with different S&S scores in the two RIF groups (Table 2). As expected, there is a higher percentage of low-RIF introns with high S&S scores (46.8% vs. 34.4% in the high-RIF group and 69.3% of introns flanking constitutive exons have S&S score > 160). However, about half of the retained introns in both RIF groups presented S&S scores in the range of 140–160 (29.3% of introns flanking constitutive exons), yielding equal odds of being retained in low or high relative frequencies. This observation further indicates that other important factors play a role in determining the frequency of intron retention.

**Table 2:** Frequency of retained introns with varying Shapiro and Senapathy splice site scores.

	% of splice sites			
S&S score (5' ss + 3' ss)	around constitutive exons	low-RIF	high-RIF	low/high-RIF ratio

80–100	0	0	0.4	0
100–119	0.1	0.3	3.6	0.1
120–139	1.4	4.1	8.4	0.5
140–159	29.3	48.8	53.2	0.9
160–179	61.1	43.8	33.2	1.3
180–200	8.2	3.0	1.2	2.5

### The influence of flanking exon and intron lengths in intron retention

**Figure 3:**
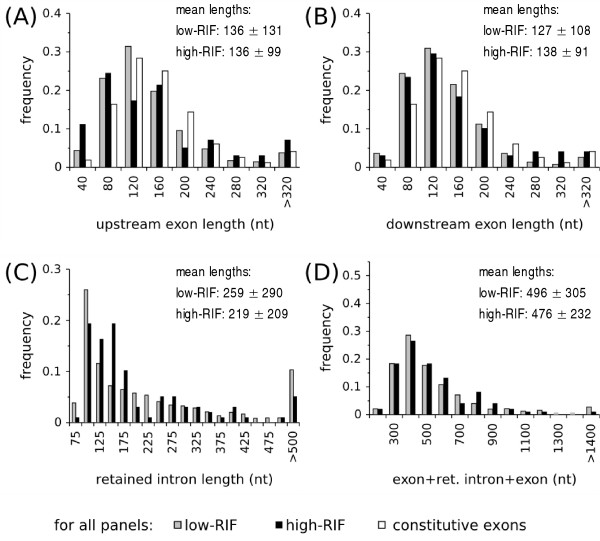
**Lengths of flanking exons, retained introns and "exon + retained intron + exon" units**. Panel (A) Distribution of lengths of upstream exons flanking the retained intron (B) Distribution of lengths of downstream exons flanking the retained intron. Data set used in (A) and (B):1193 low and 98 high-RIF exon pairs with both upstream and downstream splice borders defined by introns. (C) Distribution of lengths of retained introns. (D) Distribution of lengths of retained intron + flanking exons. Only units composed by internal exons were considered.

Based on the study of retention of a 116 nt intron flanked by 30 and 7 nt exons [9] and observations in *Drosophila *introns, Talerico and Berget [11] proposed that in vertebrates, retention would happen when introns and flanking exons are small. In the data sets analyzed here, the two exons flanking the retained intron have approximately the average length of human internal exons (~135 nt), with very few cases of very short exons (Figure 3A and 3B. Data for lengths of exons, retained and non-retained introns presented in this section are also summarized in Table S1 of Additional file 2: Lengths of retained introns and flanking exons). Also, individual examples of IR from the literature involve exons with regular lengths (Table S2 of Additional file 2: Lengths of retained introns and flanking exons). Therefore, intron retention does not seem to be related to micro-exons.

In organisms like *Drosophila *[11] and *S. pombe *[12], introns shorter than exons are recognized instead of the exons and weakening of splice sites frequently leads to intron retention. In vertebrates, IR frequently involves short introns (mean length ~100–200 nt [5,6,19,20] and individual examples in Table S2 of Additional file 2: Lengths of retained introns and flanking exons). Therefore, the length of introns is probably an important feature in the mechanism of IR, in agreement with the intron definition model. Indeed, Figure 3C shows a bias for short retained introns in both RIF groups. However, it is difficult to assess whether this trend is real using cDNA data, since ESTs bias the data for short introns. Although in [20] the authors found a difference in the length of introns retained in different frequencies, the lack of such difference in our data may be due to the fact that by requiring each event to be represented by at least 2 cDNAs, the events identified by us were those with short introns in both RIF groups that had a higher chance of being sequenced multiple times. One study that did not use ESTs to identify intron retention events, employed instead only the extent of genomic sequence conserved in human and mouse as a predictor [21]. The mean length of the 21 retained introns identified was also small (187 nt ± 156).

Although this bias for retention of short introns may also be a result of selection for inclusion of short sequences, another indirect evidence that supports a possible mechanistic relation of short introns and retention is the higher frequency of shorter *non-retained introns *in intron retaining genes. The average length of introns in our data set is 5580 nt (s.d. 17836, 120,949 introns), close to previous findings (5025 nt [22]). Genes presenting a low RIF have shorter introns (2005 ± 11797), close to the length of introns of housekeeping genes (2573), according to [22]. Non-retained introns of the high-RIF group are also shorter (3207 ± 12160, the distribution of lengths is presented in Table S3 of Additional file 2: Lengths of retained introns and flanking exons). In agreement with this observation, the mean length of non-retaining introns of the genes with intron retention identified by [21] without EST data is 3513 nt ± 8337 (166 introns, removing one gene that has an abnormally high mean intron length (LACE1, ~19,000 nt) of the 21). It is possible that this overall shorter length of introns increases the chance of a gene to undergo intron retention.

The last feature related to short introns analyzed was the size of "exon + retained intron + exon" units. Whereas the mean length of retained introns + flanking exons units is around 500 nt (Figure 3D), the mean lengths of "exon + non-retained intron + exon" units are considerably greater (all genes: 4690 nt ± 12572, n = 87824; low-RIF: 2022 nt ± 10997, n = 13486 and high-RIF: 2987 nt ± 10675, n = 2275), following the lengths of non-retained introns alone. The means are not greater than those of the corresponding non-retained introns alone, as it would be expected, because the lengths of "exon + intron + exon" units were calculated only for internal exons, excluding first introns in the 5' UTR, which are typically longer ([23] and data not shown). Figure 3D shows the distribution of lengths of "exon + retained intron + exon" units (see also Figure S1 in Additional File 2: Lengths of retained introns and flanking exons for lengths of "exon + non-retained intron + exon" units). Independently of a length bias for retention, the fact that many of the IR units are shorter than 400 nt, only slightly above the length limit for exon definition of 300 nt [13], suggests that this observation may have a mechanistic reason for at least part of the events as we discuss later.

### Genes presenting intron retention are more highly and broadly expressed than other genes

**Figure 4:**
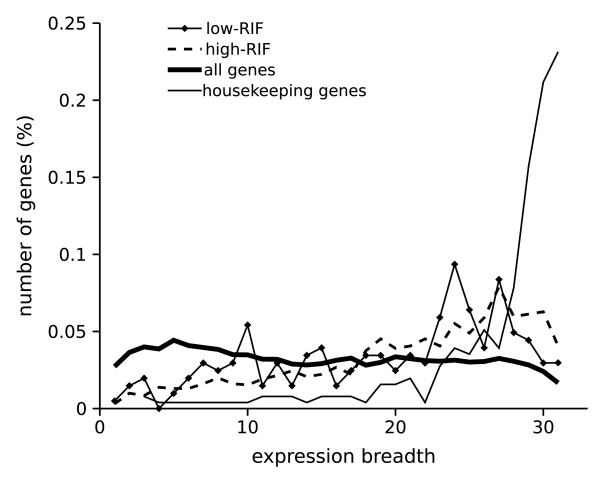
**Expression breadth (number of tissues in which a gene is expressed) of intron retaining genes**. Breadth was determined by SAGE tag counts for genes with intron retention (961 low-RIF genes, 199 high-RIF genes), all genes in the database used in this analysis (15953) and 255 out of 575 housekeeping genes (determined by [23] with microarray data). Genes presenting intron retention are more frequently broadly expressed in comparison to all genes (background distribution).

Housekeeping genes are defined as those genes that are highly expressed in most, if not all tissues and present a compact structure (less and shorter introns, [22] and references therein). Figure 4 shows that there is a bias for intron retaining genes to be expressed in many tissues in both RIF groups, indicating a slight enrichment of housekeeping genes or genes broadly expressed. In addition, housekeeping genes from [22] used here as a positive control, presented a higher expression than all genes considered together (background distribution) in all tissues available (Table S1 of Additional file 3: Analysis of gene expression). Genes with IR presented expression levels per tissue lower than housekeeping genes, but higher than all genes in 25/31 (low-RIF) and 14/31 (high-RIF) tissues (Tables S2 and S3 of Additional file 3: Analysis of gene expression).

We also observed that there is a correlation between RIF and expression levels of genes (Figure 2, panels C and D, low-RIF: ρ = -0.31, *P *< 10^-4^, high-RIF: ρ = 0.16, *P *= 0.01). Note that the correlation is negative for the low-RIF and positive for the high-RIF group. One explanation is that higher expression levels increase the amount of all transcripts, both of the minor and the major forms, but under these circumstances, very rare forms also appear. Rare splice forms in the low-RIF group will be those with strong splice sites that very rarely retain an intron, resulting in the appearance of events with low retention/non-retention ratios only with higher expression (negative correlation). The reverse would be true for the high-RIF group, in which rare forms will be those with very weak splice sites that only rarely remove the intron, and only with higher expression (positive correlation).

Such association of higher expression and RIF may be either because higher expression increases the error rate of the splicing machinery, specially because it seems that splicing occurs co-transcriptionally [24], or simply allows IR (or intron removal, in the case of the high-RIF group) to be detected since more transcripts are produced. This latter explanation is attractive because we verified that clusters that presented intron retention have more cDNAs than all other genes (for example, ~17% of the intron retaining and 6% of all other clusters, have > 400 cDNAs, χ^2^-test, 1 d.f., *P *< 10^-10^, see distribution of cluster sizes in Figure S2 of the Additional file 3: Analysis of gene expression).

### Distribution and density of splicing *cis*-regulators

*Cis*-regulatory elements (exonic splicing enhancers and silencers (ESE and ESS), intronic splicing enhancers and silencers (ISE and ISS)), play an important role in constitutive and alternative splicing by acting as binding sites for *trans*-factors that promote (enhancers) or inhibit (silencers) splicing (for review, see [1]). For example, a GAA ESE was shown to be crucial to counterbalance retention of intron D of the bovine growth hormone gene [8] and of intron 3 of the 9G8 splicing factor gene [16].

As not all motifs corresponding to *cis*-regulatory elements found in exons or introns are functional, one hypothesis is that they are present in higher densities (motifs/nt) in the sequences they act [25]. Recently, Wang et al [26] showed that a particular set of ESSs that enhance splicing of retained introns is enriched in these sequences.

**Figure 5:**
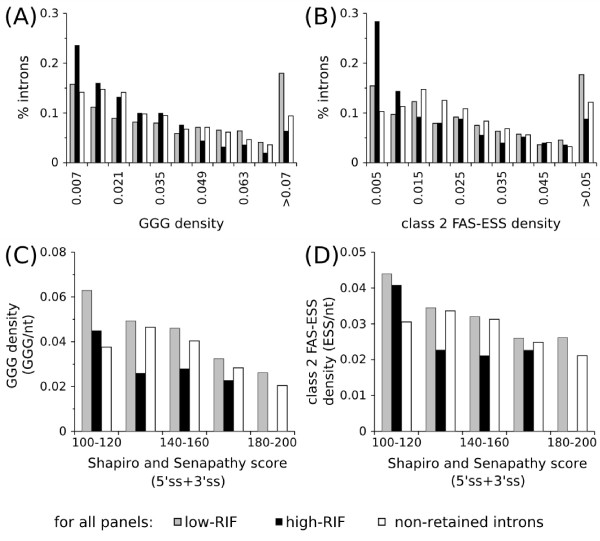
**GGG/class 2 FAS-ESS densities in retained and non-retained introns and variation with splice site strength**. Panels (A) and (B): Distribution of the frequency of retained and non-retained introns with varying densities of GGG (A) and class 2 FAS-ESSs (B). The low-RIF group presents a bias for introns with high GGG density, whereas the high-RIF group presents the opposite trend. Panels (C) and (D): Mean GGG (C) and class 2 FAS-ESSs (D) densities in non-retained and retained introns flanked by splice sites with different S&S scores. Only classes of S&S scores with more than 5 sequences were plotted.

To evaluate whether the different RIFs are associated with different densities of *cis*-regulatory elements, we scanned retained and non-retained introns, as well as exons of the same genes, for known regulatory motifs (Table 3 and see distribution of motif densities in Figure 5 and Additional File 4: Distribution of the frequency of ESE densities). The average densities of SELEX-ESEs (SF2/ASF, SC35, SRp40 and SRp55) in exons and of FAS-hex3 ESSs in retained and non-retained introns shown in Table 3 were in the range found previously ([25] and [26], respectively).

**Table 3:** Densities of putative *cis*-regulatory motifs in exons, retained and non-retained introns of genes of the low and high-RIF groups.

		introns		exons		
	*cis*-regulatory motif	retained	non-retained	flanking upstream	flanking downstream	all other

	SF2/ASF	*0.0415*^+i^	*0.0365*	0.0458	0.0451	0.0430
	SC35	0.0457^+i^	*0.0413*	0.0445	0.0450	0.0425
	SRp40	0.0388	*0.0384*	0.0399	0.0395	0.0394
low	SRp55	*0.0219*	*0.0220*	0.0272	0.0264	0.0261
	RESCUE-ESEs	*0.0442*^-i^	*0.0541*	0.0837	0.0850	0.0951
	GAA (ESE)	*0.0115*^-i^	*0.0142*	0.0191	0.0197	0.0219
	GGG (ISE)	**0.0397**^+i^	**0.0333**	0.0185	0.0190	0.0165
	FAS-ESS hex-3 class 1	**0.0047**^-i^	**0.0069**	0.0010	0.0009	0.0011
	FAS-ESS hex-3 class 2	**0.0294**^+i^	**0.0276**	0.0110	0.0115	0.0101

	SF2/ASF	*0.0383*^+i^	*0.0345*	0.0445	0.0458	0.0423
	SC35	0.0409	*0.0387*	0.0447	0.0412	0.0428
	SRp40	0.0394^+i^	*0.0371*	0.0404	0.0397	0.0408
high	SRp55	0.0231	*0.0219*	0.0256	0.0241	0.0250
	RESCUE-ESEs	*0.0548*	*0.0591*	0.0952	0.0971	0.0974
	GAA (ESE)	*0.0137*^-i^	*0.0156*	0.0227	0.0206	0.0224
	GGG (ISE)	**0.0264**^-i^	**0.0300**	0.0164	0.0173	0.0165
	FAS-ESS hex-3 class 1	**0.0043**^-i^	**0.0074**	0.0017	0.0007	0.0013
	FAS-ESS hex-3 class 2	**0.0208**^-i^	**0.0253**	0.0095	0.0106	0.0098

mean densities in italics are lower than in exons (*P *≤ 0.05), mean densities in boldface are higher than in exons (*P *≤ 0.05). The sign ^+i ^indicates that the density of the motif in retained introns is significantly higher (*P *≤ 0.05) than in non-retained introns, ^-i ^indicates it is lower.

In comparison to exons, non-retained introns were found to be poor in ESEs, in accordance to a previous study [25], and enriched with ESSs and the ISE GGG (Table 3).

Retained introns were also different from exons, but with slightly higher densities of SELEX-ESEs, intermediary to exons and non-retained introns (Table 3). This trend was not observed for RESCUE-ESEs and GAA, probably because these motifs are purine-rich (40% of RESCUE motifs have at least one GAA; notice that RESCUE-ESE and GAA data follow each other's tendencies closely). It seems that the peculiar nucleotide composition of retained introns can harbor less purine-rich motifs, but SELEX-ESEs are not affected in the same way, since they have a different nucleotide composition (see Table S5 in Additional File 5: Density of *cis*-regulatory elements in long exons).

Major differences between the low and high-RIF groups are those related to the densities of GGG and ESSs. Low-RIF retained introns presented a slightly higher density of GGG than non-retained introns, whereas high-RIF retained introns presented the opposite trend (Table 3 and Figure 5A). In regard to FAS-hex3 ESSs, Wang et al [26] showed that class 1 ESSs are actually under-represented in retained introns in relation to non-retained introns, which was also observed in our data for both RIF groups. Class 2 FAS-ESSs, however, followed the same trend of GGG, showing higher densities in the low-RIF than in non-retained introns and lower densities in the high-RIF group (Table 3 and Figure 5B). This difference between class 1 and class 2 ESSs is probably related to the fact that the latter are G-rich (5/26 class 1 ESSs and 28/46 class 2 ESS have at least one GGG triplet).

As IR will result in one long exon, being the commonest isoform of the high-RIF group, we evaluated the possibility that such differential motif distribution between flanking exons and retained introns could be actually typical of an architecture of long exons (divided in flanking segments and a central segment corresponding to the intron, *i.e*. a pseudo-retained intron), and not to retained introns themselves. Analysis of exons of length between 300–600 nt and > 600 nt showed that these sequences do not present the pattern observed for "exon + retained intron + exon" units (Additional file 5: Density of *cis*-regulatory elements in long exons). Rather, the flanking segments and pseudo-retained introns presented motif densities similar to exons in general and therefore higher than those of retained and non-retained introns, indicating that the observed differences in Table 3 are specific to introns that are retained.

The ESS/GGG densities observed could suggest that *cis*-regulatory elements play a role in differentially regulating IR in the two RIF groups. A higher ESS/GGG density in low-RIF retained introns could increase the efficiency of intron splicing (thus, lower RIFs) whereas for high-RIF introns, the inverse would be true. The basis for this argument is that knocking out different GGG triplets in the second intron of α-globin 2 leads to different levels of intron retention [10] and that inserting ESSs in the frequently retained intron 3 of the NKIRAS2 gene increased the levels of the spliced form [26]. This would explain why introns with S&S score in the range of 140–160 can be retained in both low and high RIFs (see the distribution of S&S scores in Table 2 and GGG/ESS density as a function of S&S scores in Figures 5C and 5D).

The interpretation of the slightly higher SF2/ASF and SC35 densities observed in retained introns is not straightforward. Although it might be only due to a biased nucleotide composition, another possibility is that these ESEs could help defining short "exon + retained intron + exon" units as a single exon (see Discussion), increasing retention. The other possibility is an action in the recognition of splice sites. Although ESEs are exonic by definition, SR proteins binding to introns was shown to both increase and decrease splicing in different studies (see discussion in [25,27]). If these ESEs increase intron splicing in the events analyzed here, they would counterbalance intron retention. Both cases could be happening in different intron retention events, but the fact that low-RIF densities for SF2/ASF and SC35 are higher than in high-RIF could indicate that their role is in counterbalancing intron retention. However, the signals are too subtle to allow definite conclusions to be made.

One could argue that both the higher density of SELEX-ESEs and lower ESS/GGG density in the high-RIF set of retained introns could be due to selection on sequences that occasionally are exonic, but this possibility is challenged by RESCUE-ESE and class 1 FAS-ESS, whose densities are similar to *bona fide *non-retained introns.

### Conservation of the frequency of intron retention in mouse

Evolutionary conservation of given features is often taken as a signal of biological function, under the assumption that negative selection is acting on them. Studying conserved IR events could support the observations made with human data and therefore help understanding the mechanism of intron retention. As the criteria we used to select high-confidence human IR events may have filtered out true events, in this analysis we searched the mouse genome and transcriptome for all the IR events initially detected. With the exception of the number of conserved events, all other data (identities and percentages) were the same when searching only filtered human events.

Table 4 (row 1) shows that human retained introns of the high-RIF group were almost as conserved as flanking coding exons (81% ± 9 vs. 91% ± 3) whereas introns retained in low RIF in human presented an average identity corresponding to that of homologous non-retained introns (59% ± 10 vs. 60% ± 10).

**Table 4:** Conservation of human intron retention events in the mouse.

	low-RIF	high-RIF
1. mean %ID (human retained introns versus mouse genome)^1^	59% ± 10 (1582 introns)	81% ± 9 (408 introns)
2. mean %ID (exons)	91% ± 3	91% ± 3
3. number of events presenting only the non-retaining form	1252/1297^3 ^(97%)	16/201^3 ^(8%)
4. number of events presenting only the retaining form	16/1297^3 ^(1%)	177/201^3 ^(88%)
5. number of conserved events^2^	29/1297^3 ^(2%)	8/201^3 ^(4%)
6. number of conserved events with conserved RIF	22/29	7/8
7. mean %ID of conserved retained introns with conserved RIF	86% ± 10 (22 retained introns)	91% ± 9 (7 retained introns)
8. mean %ID of non-conserved retained introns^2^	59% ± 9 (no retention in mouse; 1252 introns)	82% ± 9 (no non-retained form in mouse; 177 introns)
9. mean length of conserved retained intron	152 ± 83 (22 introns)	142 ± 87 (7 introns)
10. information content of splice sites^4^	7.2 bits ± 0.2/9.7 bits ± 0.4 (22 introns, all GT..AG)	5.4 bits ± 1.3/7.4 bits ± 1.3(7 introns, all GT..AG)

^1 ^only introns with similar lengths and borders in human and mouse were considered

^2 ^conserved in the mouse if the event presented ≥ 1 cDNA with the retained intron and ≥ 1 without. Non-conserved: only the major form was found in mouse cDNAs

^3 ^the total number of events (1297 and 201) corresponds only to those events from row 1 with a least one mouse cDNA aligned to the genomic region corresponding to the event

^4 ^with the exception of the high information content of the 3'ss of the low-RIF group, values were very similar to those observed for human

Most IR events presented only the major form in mouse (Table 4, rows 3 and 4), being considered non-conserved. Those events that presented both minor and major forms in both human and mouse were deemed conserved (29 low-RIF and 8 high-RIF). Among these events, 22/29 in the low-RIF and 7/8 in the high-RIF group presented conserved RIFs. According to our supposition, 8/22 and 2/7 of the human low and high-RIF events, respectively, had been filtered out. This indicates that our filter was stringent, removing possible true events, and considering all the events in the mouse analysis was adequate. The small percentage of events found to be conserved is probably due to the lack of cDNA data covering the homologous region, rather than lack of conservation.

Interestingly, conserved retained introns presented a high percent identity, irrespective of the RIF group (Table 4, row 7), close to that of exons in general, whereas those human IR events of the low-RIF group which presented only the major form in mouse presented the same percent identity of non-retained introns (Table 4, row 8).

As observed for human there was a difference in the information content of splice sites, those of the low-RIF group showing higher conservation (Table 4, last row, see sequence logos as Additional file 6: Sequence logos of splice sites of retained introns conserved in mouse). Irrespective of the frequency of retention, retained introns in the mouse were considerably shorter than the average non-retained introns. Data for the distribution and density of *cis*-regulatory elements are not provided for mouse conserved events due to the extremely reduced size of the data set. It is worth noting, however, that the trend of GGG/ESS density in conserved events was consistent with that of human, *i.e*. notably higher in low-RIF retained introns.

## Discussion

The main problem in analyzing IR is to guarantee that the events are authentic alternative splicing occurrences and not part of partially processed messages. However, the fact that we found a mechanistically coherent association of splice site strength and differential densities of *cis*-regulatory motifs with the two RIF groups (corroborated by conserved events) indicates that our data set contains a large fraction of authentic IR events.

In agreement with previous experimental observations on individual vertebrate retained introns [7-9,16] and with bioinformatic analyses [6,20], we found that IR is associated with weaker splice sites. We extended this general observation showing that a decrease in splice site strength leads to higher relative frequencies of retention. However, a non-negligible fraction of events bear strong splice sites. Extending a previous observation that a specific set of ESSs was able to inhibit intron retention [26], we showed that there is an inverse association of the density of these regulatory elements (and also of the ISE GGG) with the frequency of IR. The fact that a considerable fraction of low-RIF retained introns presented strong splice sites and high GGG/ESS density indicates that other factors more complex than those studied here are also involved in the regulation of this type of alternative splicing as for example the relative concentration of *trans*-factors, the formation of secondary structures in the RNA molecule or the dependence of splicing of upstream introns for retention of a specific intron, as observed for the thrombopoietin gene [7].

Although cDNA data sets like ours are possibly biased for short retained introns, other observations, not directly based on cDNA data, support the notion that such introns are short ([6,21] and see Additional file 2: Lengths of retained introns and flanking exons). Together with our observation that genes with IR present overall shorter introns these data suggest that this may have mechanistic reasons for at least part of the events. One attractive possibility is that these short retained introns are part of longer units (up to 300 nt, the limit for exon definition [13]) that are at times recognized as single long exons and at times as split "exon + intron + exon" structures. In support to this view, flanking exons seem to have the length of exons in general. In addition, the retained introns (or intra-exonic segments) contain intronic features necessary to be occasionally spliced out, such as (weak) cryptic splice sites and splicing enhancers as GGG triplets that enhance intron removal through intron definition [10] and ESSs. According to our data, in this model the relative frequency of retention would be regulated by the strength of splice sites and GGG/ESS density.

**Figure 6:**
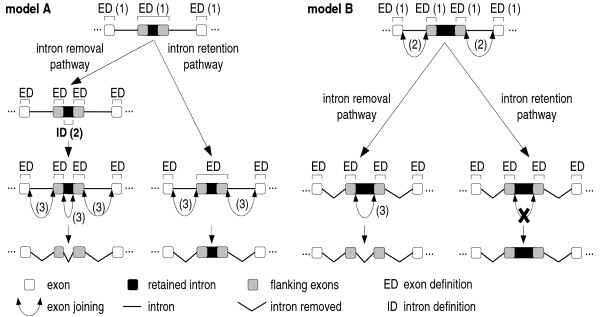
**Bipartite model of intron retention**. (A) "Two-step splice site recognition" (a mixed model of exon and intron recognition) for short introns in short "exon + intron + exon" units (< 400 nt, close to the size limit for exon recognition). In step 1, all the exons are defined, including the "exon + intron + exon" unit in black and gray. Step 2 is alternative, if intron definition occurs in the "exon + intron + exon" unit, the intron will be removed. Skipping of this step will lead to intron retention. (B) Model for long introns, typically with high scoring splice sites in long "exon + intron + exon" units. Step 1: exons are defined, including those flanking the intron to be retained (gray exons). In step 2, flanking introns are removed, joining the retained intron flanking exons to the outer exons. Step 3 is alternative, if the flanking exons are joined, the intron is removed; otherwise, abrogation of this step by hypothetical failure in E to A or A to B complex transition would lead to intron retention.

Based on this scenario, we propose a mechanism (labeled A in Figure 6) as part of a bipartite model to explain how introns are retained and spliced out. Model A was also proposed by Sterner and Berget [9] when analyzing *in vivo *intron retention of an "exon + intron + exon" sequence shorter than 300 nt from chicken troponin I with the intron 5'ss mutated. Later, Talerico and Berget [11] suggested that this would be the general mechanism for intron retention in vertebrates.

The mechanism in model A could be related to the fact that handling long exons is complicated for vertebrate spliceosomes [28] and it is tempting to speculate that the model could be extended to "exon + retained intron + exon" units longer than 300–400 nt. It was observed that an expanded exon of 566 nt could not be correctly spliced, but suffered internal splicing, creating three new small exons of 63, 90 and 73 nt and two introns shorter than 90 nt each. When expanding to 894 nt, two exons of 63 and 73 nucleotides and one intron were created [28]. These observations support the idea proposed in model A that retained introns would use weak internal cryptic splice sites that are recognized during exon definition of a long exon.

However, we also verified that there are cases where retained introns are much longer than those known to be defined and would not fit in the relatively short "exon + intron + exon" units above. For these cases, we propose an alternative, and speculative, model (mechanism B in Figure 6) that does not involve intron definition. This mechanism implies that the introns flanking the retained one should be removed first, *i.e*., intron removal order may dictate the outcome of the splicing pattern, which has already been observed to influence exon skipping [29]. Occasionally, the transition from E to A or even from A to B complex (during exon-definition to intron-bridging complex conversion), which are possible points of alternative splicing regulation [1], could suffer some kind of interruption due to the weakness of splice sites, halting later steps and retaining the intron.

Gene expression level would not be an intrinsic part of the model proposed above, but may contribute by even abolishing intron retention in tissues where expression is lower. The occurrence of more than one event per gene specially in the low-RIF group may be related to the fact that these genes present overall shorter introns, increasing the probability of an intron being retained through model A. With higher gene expression, retention of introns with moderately strong splice sites would be facilitated, though in low frequency. For introns to be retained in high frequency, splice sites would have to be considerably weaker, which due to selection are found in a minority of the introns, and therefore the chance of multiple events happening in the same gene is probably smaller.

## Conclusion

Although splice site strength is associated with a large fraction of IR events and in these cases is likely to determine whether an intron will be retained, and also in which frequency, other factors seem to be crucial in driving the regulation of this type of alternative splicing. The presence of specific regulatory motifs, by counterbalancing splice site strength, mainly for those introns that have moderately high splice sites scores seem to be very important in driving the frequency of IR. Other features as intron length and even gene expression, together with still unknown factors, also seem to play important roles in the regulation of this phenomenon.

## Methods

### Sequence data, cDNA alignment and clustering

The human genome sequence (build 35), mRNA and EST sequences (May, 2004) were downloaded from the UCSC Genome Browser [30] and cDNA clustering was performed as explained in [5,31]. Briefly, mRNAs and ESTs were aligned to the genome using SIM4 [32] to obtain alignments with splice sites precisely defined. Only the best alignment for each sequence was kept. Clustering of cDNAs was performed by comparing genomic coordinates of aligned sequences. In order to be included in the same cluster, two sequences had to present at least one similar exon/intron boundary (± 3 nt) or an overlap of at least 30 nt if the sequences only overlapped in the extremities.

### Annotation of intron retention events and data filtering

Only spliced sequences were used for alternative splicing annotation. Intron retention, exon skipping and constitutive exons were identified in clustered cDNAs by comparison of genomic coordinates with a tool developed by us [31]. An IR event was annotated for any occurrence of at least one cDNA (ESTs and mRNAs) defining an intron through the existence of its flanking exons plus at least one other spliced cDNA containing at least parts (> 1 nt) of the flanking exons and the intron (see Additional file 7: Examples of intron retention events).

In order to obtain a high confidence set of IR events, the initial sets of 6370 low and 1838 high-RIF events were filtered to 1515 and 250 events, respectively (see Additional file 8: List of low-RIF events and Additional file 9: List of high-RIF events). Only those events where both the intron retaining and intron defining forms were confirmed by at least two cDNAs each (in the case of ESTs, from different libraries) were accepted. Also, retained introns with alternative splice borders were discarded. The only exception was when the most frequent border was confirmed by > 10 cDNAs and there was only one alternative border evidenced by a single cDNA. Only the most frequent border was used for the analyses. Retained introns shorter than 50 nt were discarded.

### Splice site identification

For each IR event of the filtered data set, one representative sequence was selected to guide extraction of splice sites from the genome. To avoid misaligned splice borders, only exon/intron borders identified by SIM4 that did not present gaps or mismatches in a window of 20 nt in the exon from both borders were accepted. Only alignments with splice site directionality coherent with the alignment strand of mRNAs in the cDNA cluster were accepted. Information content of splice borders was calculated using DELILA's *rseq *[33] for positions -3 to +6 for 5'ss and for -20 to +3 for 3'ss. Sequence logos were generated using WebLogo [34].

### Gene expression measurements

The number of tissues in which a given gene is expressed (breadth) and the expression levels were determined through SAGE tag counts [35]. Short SAGE (10 nt) libraries were downloaded from SAGE Genie [36].

*Expression levels: *for one mRNA with a poly(A) tail (at least 5 consecutive As in the 3' end of the sequence) from each cDNA cluster, the 3' most NlaIII site was searched and 10 nt downstream were extracted as the bona-fide SAGE tag produced by the given gene [36]. The tag was then searched in all SAGE libraries and its frequency counted. Redundant tags (same tag for at least 2 different genes) were excluded. Tag frequencies were normalized for library size by dividing the tag frequency by the total number of tags in the library multiplied by an arbitrary factor of 200,000. Normalized counts greater than 500 were considered outliers, representing about 0.3 % of the counts.

*Comparison of gene expression levels among sets of genes: *the global expression level of a group of genes for a given tissue was taken as all normalized expression levels of all libraries available for the tissue. To compare expression levels between a given experimental group (housekeeping, low or high-RIF) and all other genes, a bootstrap-like process of data randomization was performed. Ten thousand random subsets of the size of the experimental group were generated from the set of expression levels of all genes and had their mean expression level compared to that of the experimental set. If the random expression was different than (higher or lower, depending on the tissue) that of the experimental set, the counter was increased by 1. Expression levels were considered different when at most 5% of the random subsets compared had an expression level higher (or lower, depending on the tissue) than or equal to that of the experimental set.

### Density of splicing *cis*-regulatory motifs

Putative and experimentally tested *cis*-regulatory elements from the literature were searched in exon, non-retained intron and retained intron sequences. Four scoring matrices for SF2/ASF, SC35, SRp40 and SRp55 (SELEX-ESEs) and their default cut-off scores [37] were used as sample ESEs. In addition, 238 putative ESEs [38] were scanned together as one category (RESCUE-ESEs). The ESE motif GAA [39], ESSs from [26] (FAS-hex3) and GGG, a known ISE [10] were also scanned. To normalize for length, only the initial and final 200 nt were scanned, sliding 1 nt at a time. For sequences shorter than 400 nt, the entire sequence was scanned. All occurrences were then counted and summed in their respective categories and divided by the total number of nucleotides scanned to yield motif densities (motifs/nt). The evaluation of the statistical significance of different densities in each sequence set was carried out with a bootstrap-like procedure similar to that used for gene expression measurements.

### Conservation of intron retention in mouse

*Identity of human/mouse introns and exons*: human cDNAs without the retained intron were aligned with Blast [40] (E-value = 10^-10^) to the mouse genome sequence (build 33) obtained from UCSC (May, 2004) [30] and homologous intron positions were determined. Only introns that presented similar borders (± 10 nt) and lengths (± 15%) in both organisms were accepted. Also, the hit had to present continuous cDNA coordinates, *i.e*., the two exons defining the mouse intron could have a maximum gap of 20 nt in the cDNA, to ensure the intron was not simply a long alignment gap. When multiple hits existed, the one with the lowest E-value was preferred. Introns found in mouse were further globally aligned to human retained intron sequences with *needle *[41] using default parameters and had their percent identities averaged. The same procedure was used to determine the identity of non-retained introns. The identity of exons was obtained from the alignment of flanking exons to mouse cDNAs.

*Conservation of IR events*: human cDNA blocks corresponding to "exon + retained intron + exon" segments were aligned to mouse ESTs and mRNAs. Matches corresponding to the coordinates of the retained intron plus parts of the flanking exons (at least -20 nt upstream and +20 nt downstream) were accepted as evidence of intron retention in the mouse (only spliced alignments were accepted). Matches corresponding to exon + exon where the exon/intron borders were similar by ± 10 nt were taken as evidence of the intron being excised in a given mouse cDNA. The cDNA also had to be continuous (gap < 20 nt). Splice sites were identified by aligning mouse cDNAs without the retained intron to the genome with SIM4, using the same procedure applied for human IR events.

## List of abbreviations

5'ss – 5' splice site

3'ss – 3' splice site

ESE – exonic splicing enhancer

ESS – exonic splicing silencer

FAS-ESS – fluorescence-activated screen for exonic splicing silencers

IR – intron retention

ISE – intronic splicing enhancer

nt – nucleotide

RIF – relative isoform frequency

## Authors' contributions

NJS conceived the study, proposed and performed the analyses, interpreted the data and wrote the manuscript. SJS contributed in the conception of the study, suggested analyses and revised the manuscript. All authors read and approved the final manuscript.

## Supplementary Material

Additional file 1 Comment on retained introns with non-canonical splice sites, Discussion on the presence of non-canonical splice sites in the intron retention data sets, whether they are authentic or not and how they could be recognized by the major spliceosome. Click here for file

Additional file 2 Lengths of retained introns and flanking exons, Table S1 summarizes data for lengths of exons, retained and non-retained introns, Table S2 presents data on lengths of retained introns and flanking exons of individual cases from the literature, Table S3 presents the distribution of the frequency of introns in the data sets analyzed categorized by length. Figure S1 shows the distributions of lengths of "exon + intron + exon" units for retained and non-retained introns. Click here for file

Additional file 3 Analysis of gene expression; Tables S1-S3 present the statistical analysis of gene expression per tissue of housekeeping, intron retaining genes and all other genes. Figure S1 shows that gene expression is higher for higher expression breadths, Figure S2 shows the distribution of cluster sizes of intron retaining genes in comparison to all genes. Click here for file

Additional file 4 Distribution of the frequency of ESE densities; Figure S1 displays 6 distributions of ESE frequencies (4 SELEX-ESEs, RESCUE-ESEs and GAA) for retained and non-retained introns as well as for exons and pseudo-retained introns. Click here for file

Additional file 5 Density of *cis*-regulatory elements in long exons; Table S1 shows the distribution of *cis*-regulatory elements in exons with lengths in the range of 300–600 nt and Table S2 for exons > 600 nt. Tables S3 and S4 show data for the same long exons split with an additional criterion, the presence of pseudo-splice sites. Table S5 displays data for the impact of nucleotide composition in SELEX and RESCUE-ESE densities. Click here for file

Additional file 6 Sequence logos of splice sites of retained introns conserved in mouse, Sequence logos of conserved mouse IR splice sites. Click here for file

Additional file 7 Examples of intron retention events, contains 4 figures showing examples of intron retention events in real cDNAs from the low and high-RIF groups. Click here for file

Additional file 8 List of low-RIF events, list of low-RIF IR events with GenBank accession numbers of sequences that retain/splice the intron, splice sites and RIF. Click here for file

Additional file 9 List of high-RIF events, list of high-RIF IR events with GenBank accession numbers of sequences that retain/splice the intron, splice sites and RIF. Click here for file
