# Supplementary material for: Sequence features responsible for intron retention in human
Source: BMC Genomics. 2007 Feb 26;8:59. doi: 10.1186/1471-2164-8-59 (PMC1831480; doi:10.1186/1471-2164-8-59)
Supplement: Additional file 1 — Comment on retained introns with non-canonical splice sites, Discussion on the presence of non-canonical splice sites in the intron retention data sets, whether they are authentic or not and how they could be recognized by the major spliceosome. [file 1471-2164-8-59-S1.pdf]

Additional File 1

Comment on retained introns with non-canonical splice sites

Strikingly, splice sites with up to 3 nucleotides different from G(T/C)..AG were found in the filtered high-RIF group (only 1 with 1 difference in the low-RIF). These cases are not likely to be naive alignment errors, as only perfectly aligned splice borders were used (no gaps or mismatches in 20 nt from both splice borders). Manual inspection of several cases showed that no other canonical alignment was possible. It should be noted that they were confirmed by at least one other sequence from a different library. However, they could still be due to some error in cDNA production, sequencing or due to some kind of RNA polymerase slippage, creating gaps in the cDNA and leaving random sequences in the borders to be mistakenly identified as splice sites.

Although the observation of such deviant splice sites is unexpected, non-canonical splice sites are reported to exist (Sheth et al, 2006). It is anyway intriguing how such deviant splice sites could be recognized by the spliceosome, even if rarely or erroneously. Actually, the use of non-canonical splice sites in the context of intron definition is not totally new. In *Drosophila* (Talerico and Berget, 1994) and *S. pombe* (Romfo et al, 2000), elongation of short introns caused their retention or more intriguingly, the use of sub-optimal cryptic splice sites. In the case of *S. pombe*, activation of a non-consensus GA..AG cryptic splice site occurred. Several works reported the existence of non-canonical splice sites. For example, Dong and coworkers (2000) reported a GA..GG intron in the human heparanase gene. Burset and colleagues (2000) found non-canonical splice sites in mammalian genes supported by ESTs, even after filtering the data set for wrong annotations and performing small corrections of the sequences to make them become G(T/C)..AG. As a mechanism of utilization of such deviant sites, the authors proposed that it is possible that proximal canonical splice sites are recognized, but that a 'parasitic' site nearby is actually cut. This has been experimentally shown to be the case of at least one non-canonical splice site. A GA..AG splice site has been found to be conserved in fibroblast growth factor receptors 1, 2 and 3 of human, mouse and *Xenopus* (Twigg et al, 1998) and to be correctly spliced in the presence of a closely located upstream canonical site (Brackenridge et al, 2003). As the second nucleotide in the intron may not be required for efficiency of the first catalytic step of splicing, the rest of the splice site would contain enough information for U6 recognition, more than the competing upstream GT. This way, possible relocation from the GT to GA site aided by ISEs could occur, resulting in cutting the exon/intron junction at this point (Brackenridge et al, 2003).

One other possibility for the presence of non-canonical splice sites in our data set is that these cases, mainly those with one single difference to G(T/C)..AG, are actually events of allele-specific alternative splicing (Nembaware et al, 2004). Under this assumption, the allele that generates the intron spliced form may bear G(T/C)..AG cryptic splice sites. However, as we use a single reference genome sequence supposedly bearing another allele, these sites will appear as non-canonical. This possibility can only be approached by a direct analysis of polymorphisms.

References

Brackenridge S, Wilkie AO, Screaton GR (2003) Efficient use of a 'dead-end' GA 5' splice site in the human fibroblast growth factor receptor genes. EMBO J. 22: 1620-1631.

Burset M, Seledtsov IA, Solovyev VV (2000) Analysis of canonical and non-canonical splice sites in mammalian genomes. Nucleic Acids Res. 28: 4364-4375.

Dong J, Kukula AK, Toyoshima M, Nakajima M (2000) Genomic organization and chromosome localization of the newly identified human heparanase gene. Gene 253: 171-178.

Nembaware V, Wolfe KH, Bettoni F, Kelso J, Seoighe C (2004) Allele-specific transcript isoforms in human. 577(1-2):233-238

Sheth N, Roca X, Hastings ML, Roeder T, Krainer AR, Sachidanandam R (2006) Comprehensive splice site analysis using comparative genomics. Nucleic Acid Res. 34:3955-3967.

Romfo CM, Alvarez CJ, van Heeckeren WJ, Webb CJ, Wise JA (2000) Evidence for splice site pairing via intron definition in Schizosaccharomyces pombe. Mol.Cell Biol. 20: 7955-7970.

Talerico M, Berget SM (1994) Intron definition in splicing of small Drosophila introns. Mol.Cell Biol. 14: 3434-3445.

Twigg SR, Burns HD, Oldridge M, Heath JK, Wilkie AO (1998) Conserved use of a non-canonical 5' splice site (/GA) in alternative splicing by fibroblast growth factor receptors 1, 2 and 3. Hum.Mol.Genet. 7: 685-691.
