# Supplementary material for: Sequence features responsible for intron retention in human
Source: BMC Genomics. 2007 Feb 26;8:59. doi: 10.1186/1471-2164-8-59 (PMC1831480; doi:10.1186/1471-2164-8-59)
Supplement: Additional file 2 — Lengths of retained introns and flanking exons, Table S1 summarizes data for lengths of exons, retained and non-retained introns, Table S2 presents data on lengths of retained introns and flanking exons of individual cases from the literature, Table S3 presents the distribution of the frequency of introns in the data sets analyzed categorized by length. Figure S1 shows the distributions of lengths of "exon + intron + exon" units for retained and non-retained introns. [file 1471-2164-8-59-S2.pdf]

Additional File 2

Lengths of retained introns and flanking exons

Table S1. Average lengths of retained and non-retained introns, flanking exons and all other internal exons.

	low-RIF<br>mean ± s.d. (N)	high-RIF<br>mean ± s.d. (N)
exon upstream of the retained intron[1]	136 ± 131 (1193)	136 ± 99 (98)
exon downstream of the retained intron[1]	127 ± 108 (1193)	138 ± 91 (98)
other exons not flanking a retained intron	132 ± 114 (6,736)	135 ± 106 (1120)
exon+retained intron+exon[2]	496 ± 305 (1193)	476 ± 232 (98)
exon+*non*-retained intron+exon[2, 3]	2022 ± 10997 (13,486)	2987 ± 10,675 (2275)
retained intron	259 ± 290 (1515)	219 ± 209 (250)
retained intron (EST)[4]	170 ± 112 (1114)	173 ± 103 (214)
retained intron (mRNA)[5]	506 ± 447 (401)	504 ± 396 (35)
non-retained introns[6]	2005 ± 11,797 (15,710)	3207 ± 12,160 (2758)
non-retained introns of genes with expression (SAGE) data	2051 ± 12,400 (13,701)	3207 ± 12,136 (2281)

[1] the number of events is smaller than the entire data set because only paired flanking exons that had defined upstream and downstream exon/intron borders were used to make sure the exons had been fully sequenced. Similar results were obtained projecting exon + exon coordinates on “exon + retained intron + exon” units with both borders evidenced by cDNA.
[2] only “exon + intron + exon” blocks that had defined upstream and downstream exon/intron borders were used.
[3] the mean lengths are not greater than those of introns only because first exons, which are typically longer (Hong et al, 2006), were not considered (only internal exons were used)
[4] only events reported by intron retaining ESTs
[5] only events reported by intron retaining and defining mRNAs.
[6] lengths of non-retained introns were calculated for introns defined by one mRNA (the one with more exons, to increase the data set size) per cDNA cluster (gene).

Table S2 presents the lengths of flanking exons and retained introns of genes studied experimentally. Notice that all retained introns are shorter than 5000 nt and that, with the exception of chicken troponin I, flanking exons have normal lengths.

Table S2. Lengths of individual vertebrate retained introns and flanking exons from the literature.

exon upstream	retained intron	exon downstream	exon + intron + exon	gene	reference
99	118	2219	2436	*tgif 2*	Melhuish and Wotton(2006)
81	116	469*	666	thrombopoeitin	Romano et al(2001)
162	274	303*	379	bovine growth hormone	Dirksen et al (1995)
30	116[m]	7	153	chicken troponin I	Sterner and Berget (1993)
177	875	75	1127	9G8 splicing factor	Popielarz et al (1995)
129*	148	132	409	chemokine CCL27	Ledee et al (2004)
132	205	174*	511	chemokine CCL27	Ledee et al (2004)
205	129[m]	238	472	α-globin 2	Carlo et al (1996)
290	118	137	545	kallikrein	Michael et al (2005)

notes: except where noted, the genes studied are from human. * indicates a terminal exon, [m] retained intron was only observed when 5’ss is mutated

Table S3. Distribution of lengths of non-retained introns in the data sets analyzed. Intron retaining genes present a higher frequency of shorter introns in general.

length	all genes (%)	low-RIF (%)	high-RIF (%)	Ohler et al (2005)* (%)
[illegible]	[illegible]	[illegible]	9.5	13.3
100-200	7.2	16.6	12.3	7.8
200-300	4.9	9.7	7.9	3.6
300-400	3.9	6.2	5.7	3.6
400-500	3.6	4.7	4.1	4.8
500-600	3.3	3.8	4.1	2.4
600-700	3.0	3.4	3.1	1.8
700-800	2.7	2.8	2.4	3.6
800-900	2.6	2.4	2.9	1.8
900-1000	2.5	2.3	2.4	1.8
>1000	61.2	34.1	45.5	55.4

*from data on predicted conserved intron retention events from Table 2 in Ohler et al (2005). One gene, LACE1 was excluded for being the only one with an abnormally high average intron length (~19,000 nt)

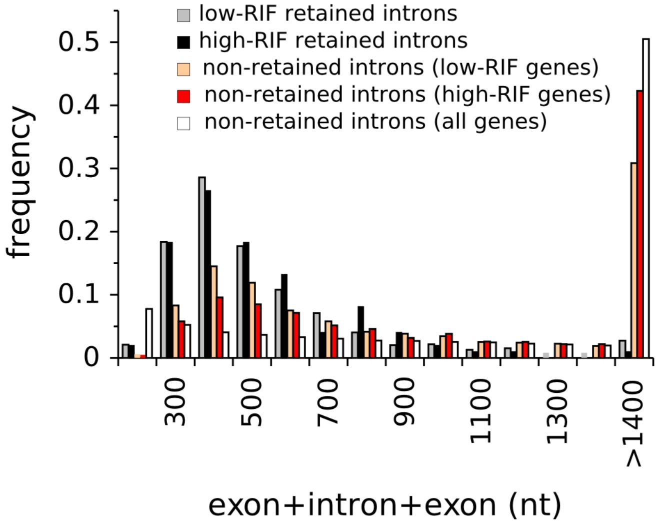

Figure S1. Distribution of lengths of exon + intron + exon units. The units containing non-retained introns were extracted from genes from the low (orange bars) and high-RIF (red bars) groups and from all genes (white bars).

References

Carlo T, Sterner DA, Berget SM (1996) An intron splicing enhancer containing a G-rich repeat facilitates inclusion of a vertebrate micro-exon. RNA. 2: 342-353.

Dirksen WP, Sun Q, Rottman FM (1995) Multiple splicing signals control alternative intron retention of bovine growth hormone pre-mRNA. J.Biol.Chem. 270: 5346-5352.

Hong X, Scofield DG, Lynch M (2006) Intron Size, Abundance, and Distribution within Untranslated Regions of Genes. Mol Biol Evol 23, 2392-2404.

Ledee DR, Chen J, Tonelli LH, Takase H, Gery I et al (2004) Differential expression of splice variants of chemokine CCL27 mRNA in lens, cornea, and retina of the normal mouse eye. Mol.Vis. 10: 663-667.

Melhuish TA, Wotton D (2006) The Tgif2 gene contains a retained intron within the coding sequence. BMC.Mol.Biol. 7: 2.

Michael IP, Kurlender L, Memari N, Yousef GM, Du D et al (2005) Intron retention: a common splicing event within the human kallikrein gene family. Clin.Chem. 51: 506-515.

Ohler U, Shorom N, Burge CB (2005) Recognition of unknown conserved alternatively spliced exons. PLoS Comput.Biol. 1: 113-122.

Popielarz M, Cavaloc Y, Mattei MG, Gattoni R, Stevenin J (1995) The gene encoding human splicing factor 9G8. Structure, chromosomal localization, and expression of alternatively processed transcripts. J.Biol.Chem. 270: 17830-17835.

Romano M, Marcucci R, Baralle FE (2001) Splicing of constitutive upstream introns is essential for the recognition of intra-exonic suboptimal splice sites in the thrombopoietin gene. Nucleic Acids Res. 29: 886-894.

Sterner DA, Berget SM (1993) In vivo recognition of a vertebrate mini-exon as an exon-intron-exon unit. Mol.Cell Biol. 13: 2677-2687.
