# Supplementary material for: Sequence features responsible for intron retention in human
Source: BMC Genomics. 2007 Feb 26;8:59. doi: 10.1186/1471-2164-8-59 (PMC1831480; doi:10.1186/1471-2164-8-59)
Supplement: Additional file 3 — Analysis of gene expression; Tables S1-S3 present the statistical analysis of gene expression per tissue of housekeeping, intron retaining genes and all other genes. Figure S1 shows that gene expression is higher for higher expression breadths, Figure S2 shows the distribution of cluster sizes of intron retaining genes in comparison to all genes. [file 1471-2164-8-59-S3.pdf]

Additional File 3

Analysis of gene expression

a. Correlation between expression levels and expression breadth

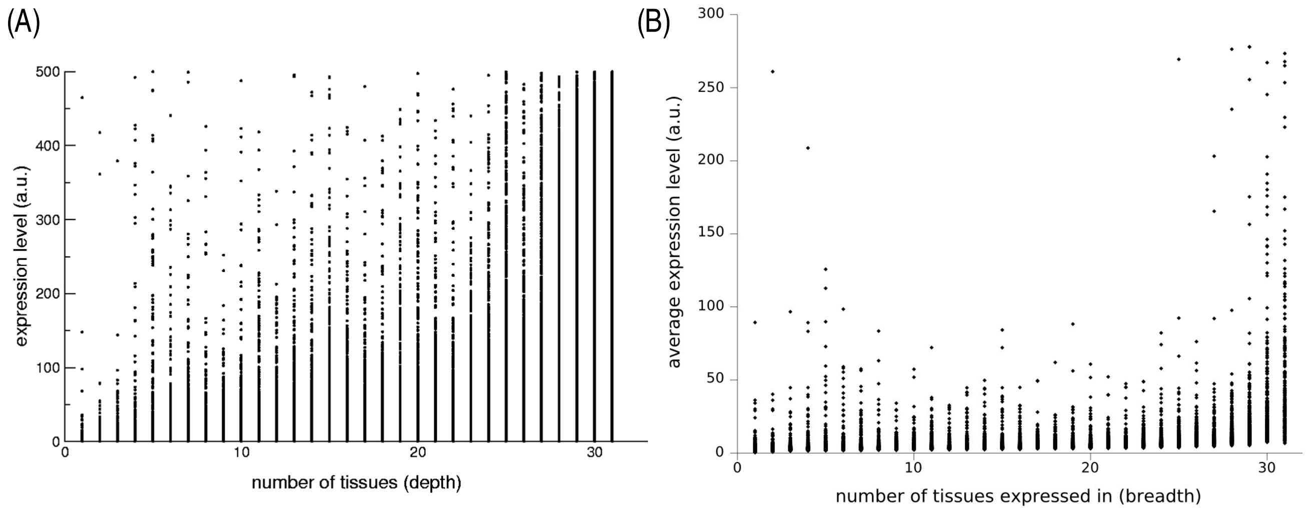

Figure S1. Correlation between gene expression level and the number of tissues they are expressed in (breadth); the higher the breadth, the higher the expression level. (A) All normalized SAGE frequencies from all libraries of all genes (15953 genes with SAGE tag in the database) in the database are plotted, (B) average frequencies of all libraries were plotted. Similar results were obtained separately for genes with low-RIF, high-RIF and housekeeping genes.

b. Analysis of global gene expression

For the analysis of gene expression levels in intron retaining, housekeeping and all genes presented in Supplementary Tables S1-S3, for a given group of genes (*e.g.* housekeeping genes), all genes in the group had their normalized expression levels determined in all libraries available for a given tissue ("n" values). The global expression level of a given group of genes for a given tissue was then taken as all "n" values together.

To compare the expression level per tissue, a bootstrap-like procedure was performed. The null hypothesis was that both the set of experimental (low and high RIF) expression levels and the set of expression levels of all genes for a given tissue had the same means. Ten thousand random subsets of the size of the experimental group were generated from the set of expression levels of all genes and had their mean expression levels compared to that of the experimental set. If the expression was different than (higher or lower, depending on the tissue) or equal to that of the experimental set, the counter was increased by 1. Expression levels of tissues were considered different when at most 5% of the random subsets compared had expression level higher than or equal that of the experimental set.

Table S1. Comparison of global expression levels (arbitrary units) estimated by SAGE (see Materials and Methods) of housekeeping genes and all genes, per tissue. The column "n" shows how many expression measurements were counted, thus each cell represents the number of genes × the number of libraries they are expressed in. Bootstrap comparisons that yielded differences in < 5% of the random sets ($P < 0.05$) are shown in red (statistically significant difference, 31/31 tissues).

	housekeeping		all genes		
Tissue	n	expression level (a.u.) mean ± sd	n	expression level (a.u.) mean ± sd	*P*-value
bone marrow	726	40.2 ± 67.6	13305	14.6 ± 31.5	$< 10^{-4}$
bone	160	43.0 ± 67.2	3370	15.8 ± 36.5	$< 10^{-4}$
brain	13463	34.0 ± 56.4	332771	12.0 ± 26.9	$< 10^{-4}$
cartilage	1853	35.3 ± 58.3	38533	13.6 ± 32.4	$< 10^{-4}$
cerebellum	4807	34.4 ± 58.8	110861	12.5 ± 27.3	$< 10^{-4}$
colon	2313	40.8 ± 70.4	46196	13.6 ± 30.0	$< 10^{-4}$
eye	222	32.4 ± 62.5	5395	9.7 ± 26.9	$< 10^{-4}$
gastrointestinal tract	167	23.1 ± 36.7	3081	11.4 ± 23.5	$< 10^{-4}$
heart	191	38.2 ± 66.3	4476	13.2 ± 34.5	$< 10^{-4}$
kidney	751	40.6 ± 62.9	14503	15.0 ± 29.8	$< 10^{-4}$
liver	793	30.3 ± 52.8	16638	13.1 ± 29.6	$< 10^{-4}$
lung	1374	37.7 ± 63.0	29168	14.3 ± 31.6	$< 10^{-4}$
lymph node	206	24.6 ± 53.7	6340	9.2 ± 21.8	$< 10^{-4}$
mammary gland	8567	34.1 ± 55.6	181819	14.4 ± 31.0	$< 10^{-4}$
muscle	348	32.2 ± 54.7	5883	15.0 ± 35.6	$< 10^{-4}$
other	198	25.9 ± 59.7	4779	10.0 ± 26.8	$< 10^{-4}$
ovary	1583	42.8 ± 64.6	31301	16.5 ± 32.1	$< 10^{-4}$
pancreas	1547	42.1 ± 67.7	27007	18.9 ± 35.2	$< 10^{-4}$
peritoneum	387	34.5 ± 64.8	7428	15.9 ± 33.4	$< 10^{-4}$
placenta	384	28.5 ± 57.5	9916	10.4 ± 26.7	$< 10^{-4}$
prostate	2802	41.2 ± 66.7	55032	15.2 ± 32.9	$< 10^{-4}$
retina	634	25.2 ± 46.9	16620	10.2 ± 24.1	$< 10^{-4}$
skin	410	38.1 ± 64.7	7118	19.6 ± 37.7	$< 10^{-4}$
spinal cord	197	32.7 ± 48.5	4791	13.6 ± 27.2	$< 10^{-4}$
stem cell	2555	39.0 ± 67.8	81368	8.7 ± 25.5	$< 10^{-4}$
stomach	1837	34.2 ± 57.8	41658	13.4 ± 29.1	$< 10^{-4}$
thyroid	603	30.7 ± 61.3	17463	9.7 ± 25.8	$< 10^{-4}$
uncharacterized tissue	353	41.9 ± 60.6	6578	19.1 ± 36.6	$< 10^{-4}$
uterus	131	35.0 ± 58.9	1695	22.1 ± 37.0	0.0022
vascular	1187	36.8 ± 66.2	26377	13.2 ± 31.6	$< 10^{-4}$
white blood cells	2265	38.3 ± 66.3	46703	14.2 ± 31.2	$< 10^{-4}$

Table S2. Comparison of global expression levels (arbitrary units) estimated by SAGE (see Materials and Methods) of genes with low-RIF events and all genes, per tissue. The column "n" shows how many expression measurements were counted, thus each cell represents the number of genes × the number of libraries they are expressed. Bootstrap comparisons that yielded differences in < 0.05 random sets ($P < 0.05$) are shown in red (statistically significant difference, 25/31 tissues).

	low-RIF		all genes		
Tissue	n	expression level (a.u.) mean ± sd	n	expression level (a.u.) mean ± sd	*P*-value
bone marrow	1463	17.9 ± 38.3	13305	14.6 ± 31.5	0.0007
bone	413	19.0 ± 36.9	3370	15.8 ± 36.5	0.0608
brain	30432	14.5 ± 33.1	332771	12.0 ± 26.9	$< 10^{-4}$
cartilage	3871	15.9 ± 35.4	38533	13.6 ± 32.4	0.0002
cerebellum	10303	15.6 ± 35.5	110861	12.5 ± 27.3	$< 10^{-4}$
colon	5057	16.3 ± 35.6	46196	13.6 ± 30.0	$< 10^{-4}$
eye	516	12.1 ± 31.9	5395	9.7 ± 26.9	0.0401
gastrointestinal tract	303	13.4 ± 28.7	3081	11.4 ± 23.5	0.111
heart	412	15.2 ± 33.7	4476	13.2 ± 34.5	0.1441
kidney	1496	17.9 ± 35.3	14503	15.0 ± 29.8	0.0011
liver	1545	15.7 ± 35.9	16638	13.1 ± 29.6	0.0014
lung	2987	17.0 ± 37.2	29168	14.3 ± 31.6	$< 10^{-4}$
lymph node	551	11.5 ± 27.2	6340	9.2 ± 21.8	0.0154
mammary gland	18323	17.7 ± 36.9	181819	14.4 ± 31.0	$< 10^{-4}$
muscle	635	14.9 ± 35.5	5883	15.0 ± 35.6	0.4899
other	490	12.4 ± 25.5	4779	10.0 ± 26.8	0.0427
ovary	3217	19.4 ± 38.1	31301	16.5 ± 32.1	$< 10^{-4}$
pancreas	3047	22.5 ± 42.0	27007	18.9 ± 35.2	$< 10^{-4}$
peritoneum	756	20.8 ± 42.2	7428	15.9 ± 33.4	0.0005
placenta	817	11.8 ± 24.2	9916	10.4 ± 26.7	0.079
prostate	5818	18.1 ± 37.9	55032	15.2 ± 32.9	$< 10^{-4}$
retina	1474	13.5 ± 28.7	16620	10.2 ± 24.1	$< 10^{-4}$
skin	730	23.7 ± 47.6	7118	19.6 ± 37.7	0.0077
spinal cord	440	14.6 ± 25.5	4791	13.6 ± 27.2	0.2378
stem cell	7102	11.2 ± 32.6	81368	8.7 ± 25.5	$< 10^{-4}$
stomach	3858	16.5 ± 37.5	41658	13.4 ± 29.1	$< 10^{-4}$
thyroid	1481	11.2 ± 28.5	17463	9.7 ± 25.8	0.03
uncharacterized tissue	658	25.1 ± 55.3	6578	19.1 ± 36.6	0.0011
uterus	192	31.7 ± 52.6	1695	22.1 ± 37.0	0.0043
vascular	2547	15.4 ± 35.3	26377	13.2 ± 31.6	0.0012
white blood cells	4970	18.3 ± 39.6	46703	14.2 ± 31.2	$< 10^{-4}$

Table S3. Comparison of global expression levels (arbitrary units) estimated by SAGE (see Materials and Methods) of genes with high-RIF events and all genes, per tissue. The column "n" shows how many expression measurements were counted, thus each cell represents the number of genes × the number of libraries they are expressed. Bootstrap comparisons that yielded differences in < 0.05 random sets ($P < 0.05$) are shown in red (statistically significant difference, 14/31 tissues).

	high-RIF		all genes		
Tissue	n	expression level (a.u.) mean ± sd	n	expression level (a.u.) mean ± sd	*P*-value
bone marrow	300	20.1 ± 37.3	13305	14.6 ± 31.5	0.0058
bone	55	22.4 ± 34.5	3370	15.8 ± 36.5	0.1039
brain	6188	13.3 ± 29.7	332771	12.0 ± 26.9	0.0004
cartilage	762	13.6 ± 25.9	38533	13.6 ± 32.4	0.507
cerebellum	2153	16.7 ± 36.1	110861	12.5 ± 27.3	$< 10^{-4}$
colon	923	16.4 ± 33.4	46196	13.6 ± 30.0	0.0053
eye	109	10.1 ± 17.7	5395	9.7 ± 26.9	0.3627
gastrointestinal tract	67	9.8 ± 10.6	3081	11.4 ± 23.5	0.3263
heart	81	12.1 ± 18.4	4476	13.2 ± 34.5	0.4383
kidney	298	18.4 ± 33.1	14503	15.0 ± 29.8	0.0342
liver	330	14.0 ± 28.6	16638	13.1 ± 29.6	0.275
lung	538	15.9 ± 32.8	29168	14.3 ± 31.6	0.1352
lymph node	107	10.8 ± 19.9	6340	9.2 ± 21.8	0.1976
mammary gland	3561	18.1 ± 40.5	181819	14.4 ± 31.0	$< 10^{-4}$
muscle	118	10.7 ± 15.1	5883	15.0 ± 35.6	0.0756
other	97	11.9 ± 18.0	4779	10.0 ± 26.8	0.2227
ovary	644	20.4 ± 41.8	31301	16.5 ± 32.1	0.0036
pancreas	549	21.3 ± 38.4	27007	18.9 ± 35.2	0.0655
peritoneum	145	20.7 ± 46.1	7428	15.9 ± 33.4	0.0585
placenta	197	10.4 ± 16.7	9916	10.4 ± 26.7	0.4504
prostate	1094	18.5 ± 42.5	55032	15.2 ± 32.9	0.0014
retina	308	13.1 ± 34.9	16620	10.2 ± 24.1	0.0327
skin	153	18.9 ± 33.4	7118	19.6 ± 37.7	0.4424
spinal cord	89	13.0 ± 26.3	4791	13.6 ± 27.2	0.4786
stem cell	1453	15.1 ± 38.7	81368	8.7 ± 25.5	$< 10^{-4}$
stomach	803	13.9 ± 23.3	41658	13.4 ± 29.1	0.2995
thyroid	306	12.4 ± 33.9	17463	9.7 ± 25.8	0.0466
uncharacterized tissue	133	25.4 ± 41.4	6578	19.1 ± 36.6	0.0391
uterus	34	30.0 ± 69.0	1695	22.1 ± 37.0	0.1286
vascular	495	16.4 ± 36.2	26377	13.2 ± 31.6	0.0251
white blood cells	929	16.0 ± 32.5	46703	14.2 ± 31.2	0.047

c. cDNA clusters that present intron retention have more sequences

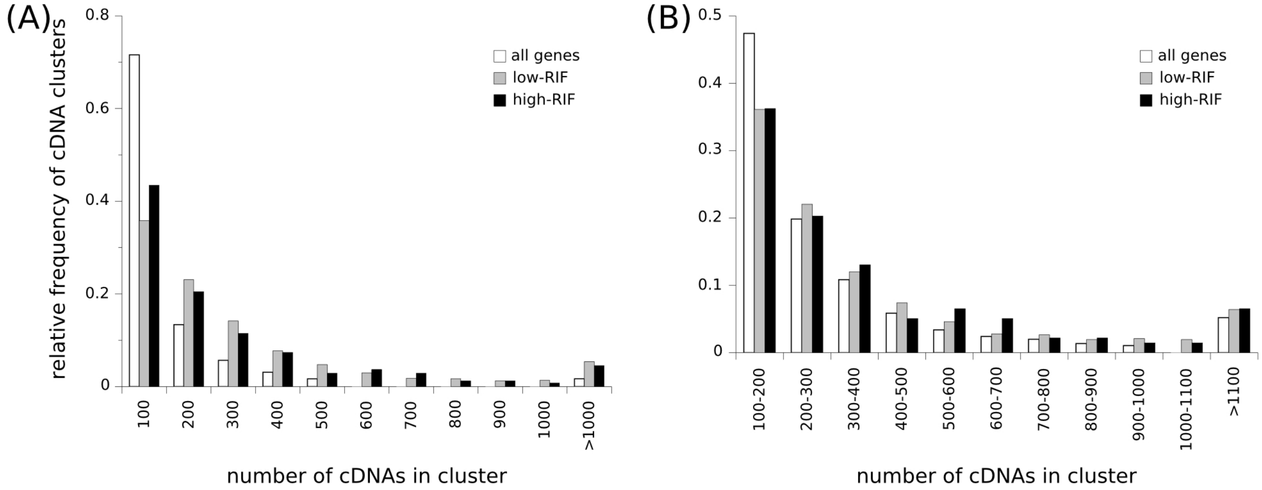

Figure S2 – Distribution of the number of cDNAs in low-RIF, high-RIF and all clusters. (A) Clusters presenting intron retention are larger. (B) The difference is not due to a paucity of small clusters showing intron retention, but still exists even considering only clusters with >100 cDNAs.
