# Supplementary material for: Sequence features responsible for intron retention in human
Source: BMC Genomics. 2007 Feb 26;8:59. doi: 10.1186/1471-2164-8-59 (PMC1831480; doi:10.1186/1471-2164-8-59)
Supplement: Additional file 4 — Distribution of the frequency of ESE densities; Figure S1 displays 6 distributions of ESE frequencies (4 SELEX-ESEs, RESCUE-ESEs and GAA) for retained and non-retained introns as well as for exons and pseudo-retained introns. [file 1471-2164-8-59-S4.pdf]

Additional File 4

Distribution of the frequency of ESE densities

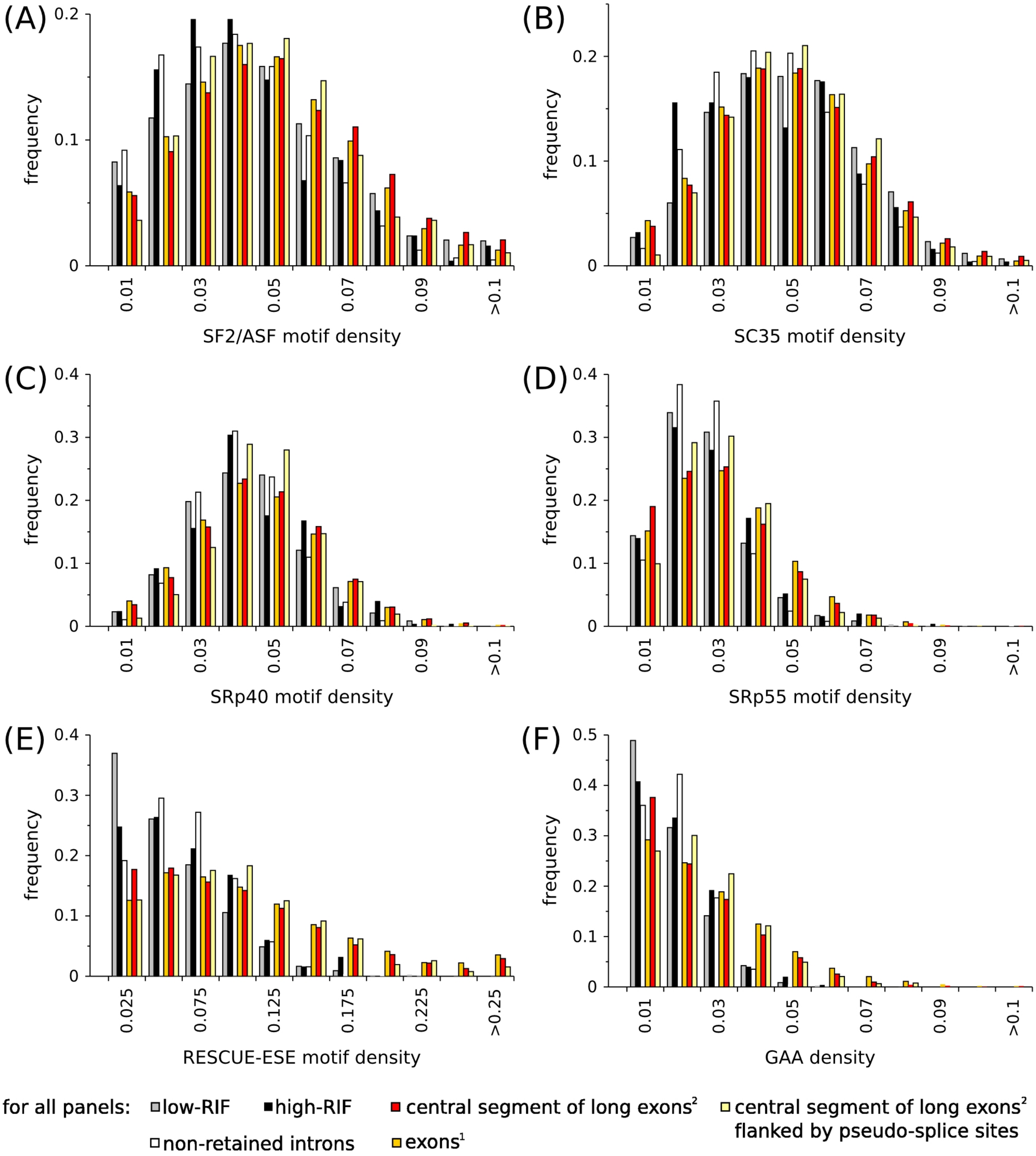

(legend in next page)

Figure S1. Distribution of ESE motif densities in exon and intron data sets.
The pattern of distribution of SELEX-ESE (Liu et al (1998), Liu et al (2000)) densities (A-D) differs from that of RESCUE-ESEs (Fairbrother et al, 2002, E) and GAA (F). This may be due to the fact that RESCUE-ESEs and GAA are purine-rich. SELEX-ESE densities (A-D) of retained and non-retained introns are not very different from those of exons, but the intron data sets seem to be slightly enriched with sequences with lower ESE densities, specially SF2/ASF (A) and SRp55 (D). In all panels, the central segment of long exons (pseudo-retained introns) and exons in general present very similar distributions of ESE frequencies, indicating that the differences observed in low and high-RIF retained introns are not related to a particularity of long exon architectures (see main text and Additional File 5).
[1] "Exons" correspond to the sets "all other exons" from the low and high-RIF groups in Table 3 together (7856 exons).
[2] The "central segments" or pseudo-retained introns were extracted from exons with length >300 and <600 nt (3207 exon segments, see main text and Additional File 5).

References

Fairbrother WG, Yeh RF, Sharp PA, Burge CB (2002) Predictive identification of exonic splicing enhancers in human genes. Science 297: 1007-1013.

Liu HX, Chew SL, Cartegni L, Zhang MQ, Krainer AR (2000) Exonic splicing enhancer motif recognized by human SC35 under splicing conditions. Mol Cell Biol 20: 1063-1071.

Liu HX, Zhang M, Krainer AR (1998) Identification of functional exonic splicing enhancer motifs recognized by individual SR proteins. Genes Dev 12: 1998-2012.
