# Supplementary material for: Sequence features responsible for intron retention in human
Source: BMC Genomics. 2007 Feb 26;8:59. doi: 10.1186/1471-2164-8-59 (PMC1831480; doi:10.1186/1471-2164-8-59)
Supplement: Additional file 5 — Density of cis-regulatory elements in long exons; Table S1 shows the distribution of cis-regulatory elements in exons with lengths in the range of 300–600 nt and Table S2 for exons > 600 nt. Tables S3 and S4 show data for the same long exons split with an additional criterion, the presence of pseudo-splice sites. Table S5 displays data for the impact of nucleotide composition in SELEX and RESCUE-ESE densities. [file 1471-2164-8-59-S5.pdf]

Additional File 5

Density of *cis*-regulatory elements in long exons

Retention of introns will generate one long exon (exon + retained intron + exon). We evaluated the possibility that the differential distribution of *cis*-regulatory elements found between retained introns and (flanking) exons could be due to a pattern common to long exons (> 300 nt), rather than to features of retained introns, specially for the high-RIF group, where the most abundant form is the one that retains the intron. For this purpose, we divided long exons (> 300 and < 600 nt, mean lengths: 135 ± 28 (Table S1) and >600 nt, mean lengths: 400 ± 250 (Table S2)) in three consecutive segments (one corresponding to the putative upstream flanking exon, another to the putative retained intron (pseudo-retained intron) and another to the putative downstream flanking exon) and compared the densities of regulatory elements among them.

The fact that the middle segment presents slightly less RESCUE-ESEs is consistent with the fact that these motifs were specifically identified by selecting over-represented hexamers close to splice borders (Fairbrother et al, 2002). In any case, the slightly different densities between the 3 segments is very far from those observed in the retained intron sets.

Table S1. Density of *cis*-splicing regulatory elements in 3207 long exons (> 300 nt and < 600 nt) divided in three segments. The three segments have similar motif densities.

cis-regulatory element	5' segment	central segment)	3' segment
SF2/ASF	0.0450	0.0461[3]	0.0443
SC35	0.0440	0.0447[3]	0.0431
SRp40	0.0424	0.0413[5]	0.0417
SRp55	0.0250	0.0250	0.0257
RESCUE-ESEs	0.0924	0.0870[5,3]	0.0950
GAA (ESE)	0.0206	0.0191[5,3]	0.0212
GGG (ISE)	0.0173	0.0200[5,3]	0.0175
FAS-ESS hex-3 class 1	0.0015	0.0016	0.0015
FAS-ESS hex-3 class 2	0.0107	0.0116	0.0104

[5] $P \leq 0.05$ in relation to the 5' segment
[3] $P \leq 0.05$ in relation to the 3' segment

Table S2. Density of *cis*-splicing regulatory elements in 2570 long exons (> 600 nt) divided in three segments. The three segments have similar motif densities.

cis-regulatory element	5' segment	central	3' segment
SF2/ASF	0.0427	0.0424	0.0425
SC35	0.0427	0.0427	0.0420
SRp40	0.0428	0.0421	0.0423
SRp55	0.0250	0.0245	0.0244
RESCUE-ESEs	0.0968	0.0890[5,3]	0.0929[5]
GAA (ESE)	0.0219	0.0201[5,3]	0.0210[5]
GGG (ISE)	0.0175	0.0197[5]	0.0187[5]
FAS-ESS hex-3 class 1	0.0028	0.0034[5]	0.0032[5]
FAS-ESS hex-3 class 2	0.0118	0.0135[5]	0.0130[5]

[5] $P \leq 0.05$ in relation to the 5' segment
[3] $P \leq 0.05$ in relation to the 3' segment

Selection of pseudo-retained introns flanked by pseudo-splice sites

Table S3 shows long exons >300 nt and <600 nt and Table S4 exons >600 nt split in a different way than that of Tables S1 and S2. Each long exon was additionally scanned for the presence of a GT 5'ss (-3 to +5) and an AG 3'ss (-20 to +1), scoring at least 60 each in the S&S scoring system (120 was the minimum score observed in IR data sets), in order to verify whether the presence of such sites in long exons could be the cause of the differential motif densities in retained-introns.

- the GT should be at a distance > (1/3 of the exon length - 30% of the exon length) from the long exon's start
- the AG should be at a distance > (1/3 of the exon length + a randomly chosen distance of maximum 1/2 of the exon length) from the GT, but at least 100 nt before the end of the long exon

All sequences not complying with these restraints were discarded.

The sequence before the GT was considered the pseudo-upstream flanking exon (5' segment), the sequence between the GT and the AG was considered the pseudo-retained intron (central segment) and the sequence after the AG was considered the pseudo-downstream flanking exon (3' segment).

The parameters above were varied for testing and the results were fairly stable overall. This specific set of parameters was chosen to yield similar average lengths of pseudo-flanking exons (Table S3: 5' segment: 132 ± 32, pseudo-retained intron: 199 ± 46 and 3' segment: 135 ± 27, Table S4: 5' segment: 356 ± 202, pseudo-retained intron: 545 ± 336, 3' segment: 385 ± 265).

The presence of pseudo-splice sites does not affect the density across long exons divided in pseudo-retained introns and pseudo-flanking exons

The three segments were scanned for regulatory motifs and the results are presented below. They show that requiring the presence of pseudo-splice sites did not alter our previous conclusions. The three segments have similar motif densities, close to those of exons, indicating that retained introns have a differential motif density that is not simply due to the architecture of “exon + intron + exon” units. Differences are probably intrinsic to the sequences, relying on their nucleotide compositions.

Table S3. Density of *cis*-splicing regulatory elements in 755 long exons (> 300 nt and <600 nt) divided in three segments with GT..AG pseudo-splice sites flanking the central segment. The three segments have similar motif densities.

cis-regulatory element	5' segment	central	3' segment
SF2/ASF	0.0428	0.0429	0.0424
SC35	0.0424	0.0447[5,3]	0.0419
SRp40	0.0428	0.0413	0.0414
SRp55	0.0245	0.0249	0.0252
RESCUE-ESEs	0.0960	0.0872[5,3]	0.0955
GAA (ESE)	0.0215	0.0197	0.0215
GGG (ISE)	0.0156	0.0188[5,3]	0.0155
FAS-ESS hex-3 class 1	0.0018	0.0019	0.0015
FAS-ESS hex-3 class 2	0.0099	0.0127[5,3]	0.0098

[5] $P \leq 0.05$ in relation to the 5' segment
[3] $P \leq 0.05$ in relation to the 3' segment

Table S4. Density of *cis*-splicing regulatory elements in 2255 long exons (> 600 nt) divided in three segments with GT..AG pseudo-splice sites flanking the central segment. The three segments have similar motif densities.

cis-regulatory element	5' segment	central	3' segment
SF2/ASF	0.0420	0.0408	0.0414
SC35	0.0420	0.0418	0.0409
SRp40	0.0427	0.0415[5]	0.0419
SRp55	0.0243	0.0245	0.0238
RESCUE-ESEs	0.1009[5,3]	0.0903	0.0955
GAA (ESE)	0.0228[5,3]	0.0207	0.0218
GGG (ISE)	0.0170[5]	0.0194[3]	0.0178
FAS-ESS hex-3 class 1	0.0024[5,3]	0.0040	0.0030
FAS-ESS hex-3 class 2	0.0109[5,3]	0.0142[3]	0.0126

[5] $P \leq 0.05$ in relation to the 5' segment
[3] $P \leq 0.05$ in relation to the 3' segment

The presence of pseudo-splice sites affects the overall density of ESEs, as a result of selecting sequences with a biased nucleotide composition

Notice that SELEX-ESE densities in Tables S3 and S4 are lower than those in Tables S1 and S2. Note that in all analyses the differences were always homogeneous among the 3 segments, with no bias for the pseudo-retained intron segment. This difference can be explained by the fact that requirement of the presence of pseudo-splice sites scoring >60, resulted in the selection of sequences with a specific nucleotide composition. Such composition allowed the appearance of pseudo-splice sites and also caused a decrease in ESE densities.

To assess the veracity of this proposal, we tested the impact of different pseudo-splice sites (data not shown). Even the presence of pseudo-splice sites as TT..TT or AA..AA had a similar impact on SELEX-ESE densities. Therefore, the differences were probably not due to the presence of splice sites, but because sequences with a nucleotide composition slightly different from the average were selected (in the cited case, probably richer in T or A).

In addition, the set of discarded sequences (without the pseudo-splice sites) presented the opposite trend, *i.e.* very high SELEX-ESE densities, confirming that it is the selection of sequences with a biased nucleotide composition that affects motif densities.

Biased nucleotide compositions have different effects in RESCUE-ESE/GAA and SELEX-ESE densities

Whereas SELEX-ESEs suffered a slight decrease in the analysis above, related to a biased nucleotide composition, RESCUE-ESE/GAA densities had almost no variation (Tables S1/S3 and S2/S4). This reinforces the idea that variations in nucleotide composition affects SELEX and RESCUE-ESEs/GAA differently, which explains the trends observed in Table 3 of the paper.

This is further confirmed by the fact that when we required the presence of “pseudo-splice sites” CC..CC, with a T-rich tract in the “3'ss”, that is, AG depleted exons, we coherently obtained very high SELEX-ESE motif densities and very low RESCUE-ESE densities (the lowest obtained in any set, Table S5).

This conclusion is supported by analysis of two sets of randomly generated sequences (500 sequences of 200 nt length), one with 60% AT composition and another with 60% CT (Table S5). Like the data above, depletion of AG caused decrease in RESCUE-ESEs/GAA and increase in SELEX-ESE densities (with the exception of SF2/ASF, which might suggest that other factors than nucleotide composition only play a role in establishing functional ESE densities).

Therefore, the differences between the trends of RESCUE-ESEs/GAA and SELEX-ESEs observed in Table 3 of the paper are likely to be due to the AG richness of the former set of enhancers and a biased nucleotide composition of retained introns.

Table S5. Densities of ESE motifs in sequences with different nucleotide composition bias. In bold, the highest densities between CT and AG-rich sets.

	long exons		randomly generated sequences	
cis-regulatory element	CC..CC+polyT (CT-rich)	AA..AA+polyG (AG-rich)	CT-rich	AG-rich
SF2/ASF	**0.0488**	0.0419	0.0333	**0.0481**
SC35	**0.0481**	0.0405	**0.0402**	0.0322
SRp40	**0.0443**	0.0417	**0.0427**	0.0347
SRp55	**0.0266**	0.0234	**0.0334**	0.0280
RESCUE-ESEs	0.0743	**0.1071**	0.0303	**0.0953**
GAA (ESE)	0.0160	**0.0224**	0.0077	**0.0265**

References

Fairbrother WG, Yeh RF, Sharp PA, Burge CB (2002) Predictive identification of exonic splicing enhancers in human genes. Science 297: 1007-1013.
