# Supplementary figures and images for: Sequence features responsible for intron retention in human
Source: BMC Genomics. 2007 Feb 26;8:59. doi: 10.1186/1471-2164-8-59 (PMC1831480; doi:10.1186/1471-2164-8-59)

### Sequence logos of splice sites of retained introns conserved in mouse

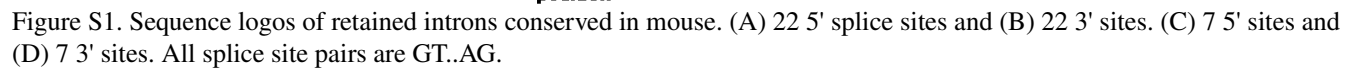

Supplement: Additional file 6 — Sequence logos of splice sites of retained introns conserved in mouse, Sequence logos of conserved mouse IR splice sites. [file 1471-2164-8-59-S6.pdf]
