# Supplementary material for: Sequence features responsible for intron retention in human
Source: BMC Genomics. 2007 Feb 26;8:59. doi: 10.1186/1471-2164-8-59 (PMC1831480; doi:10.1186/1471-2164-8-59)
Supplement: Additional file 7 — Examples of intron retention events, contains 4 figures showing examples of intron retention events in real cDNAs from the low and high-RIF groups. [file 1471-2164-8-59-S7.pdf]

Additional File 7

Examples of intron retention events

Low-RIF

(A)

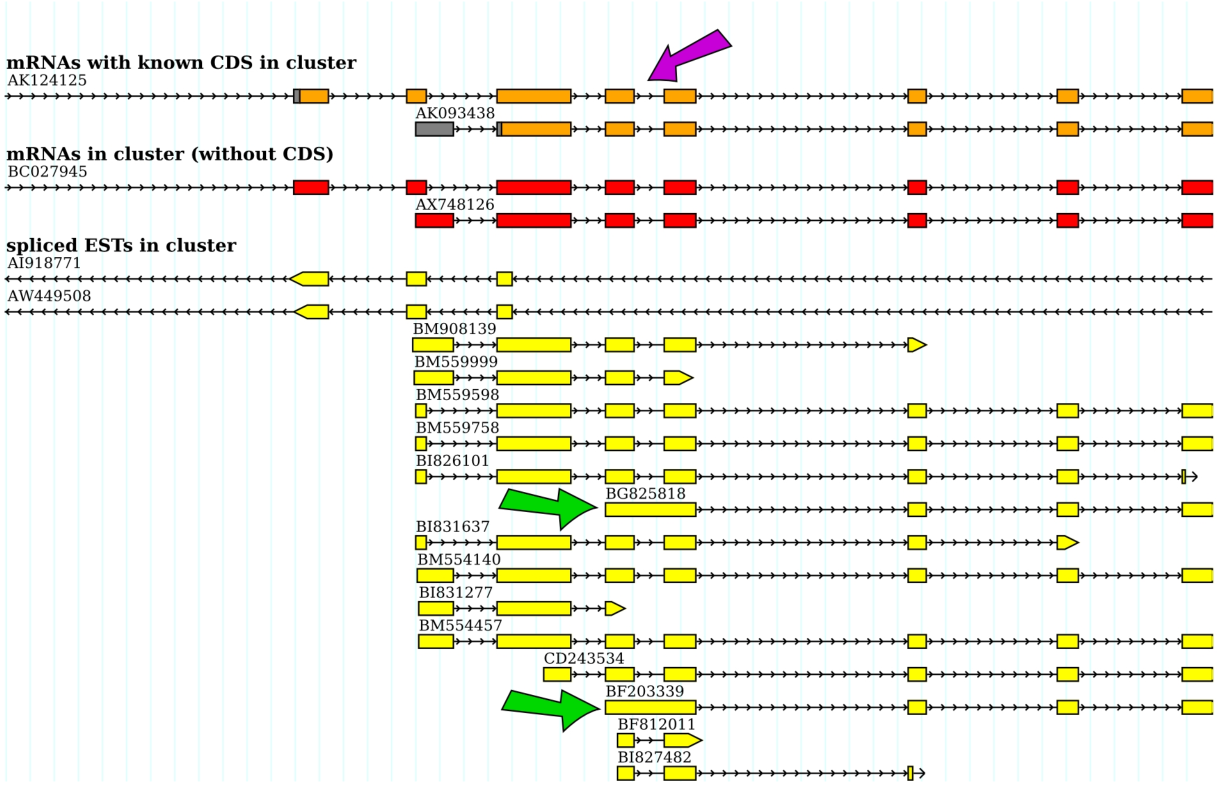

(B)

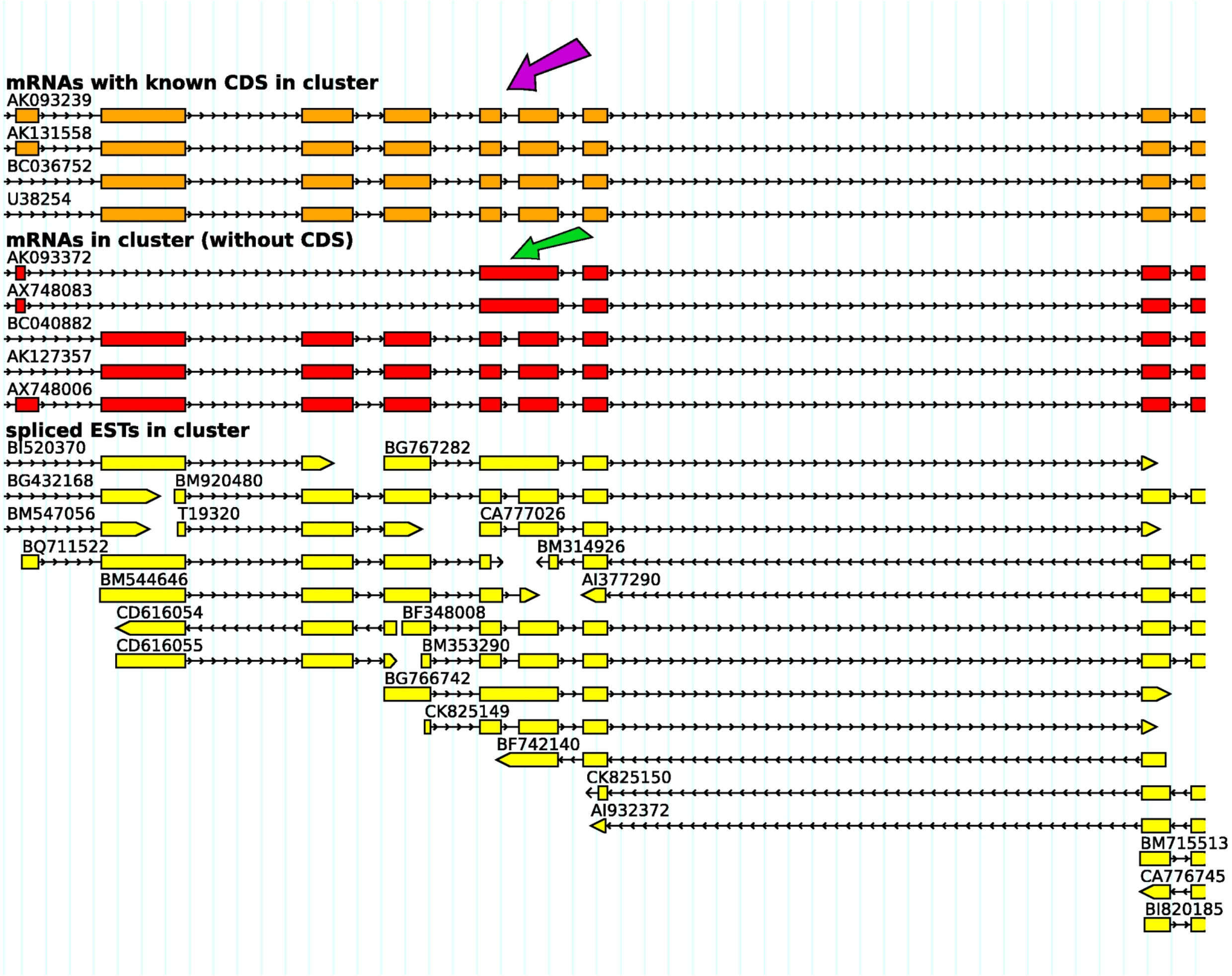

mRNAs with known CDS in cluster
AK093239
AK131558
BC036752
U38254
mRNAs in cluster (without CDS)
AK093372
AX748083
BC040882
AK127357
AX748006
spliced ESTs in cluster
BI520370
BG767282
BG432168
BM920480
BM547056
T19320
CA777026
BQ711522
BM314926
BM544646
AI377290
CD616054
BF348008
CD616055
BM353290
BG766742
CK825149
BF742140
CK825150
AI932372
BM715513
CA776745
BI820185

High-RIF

(C)

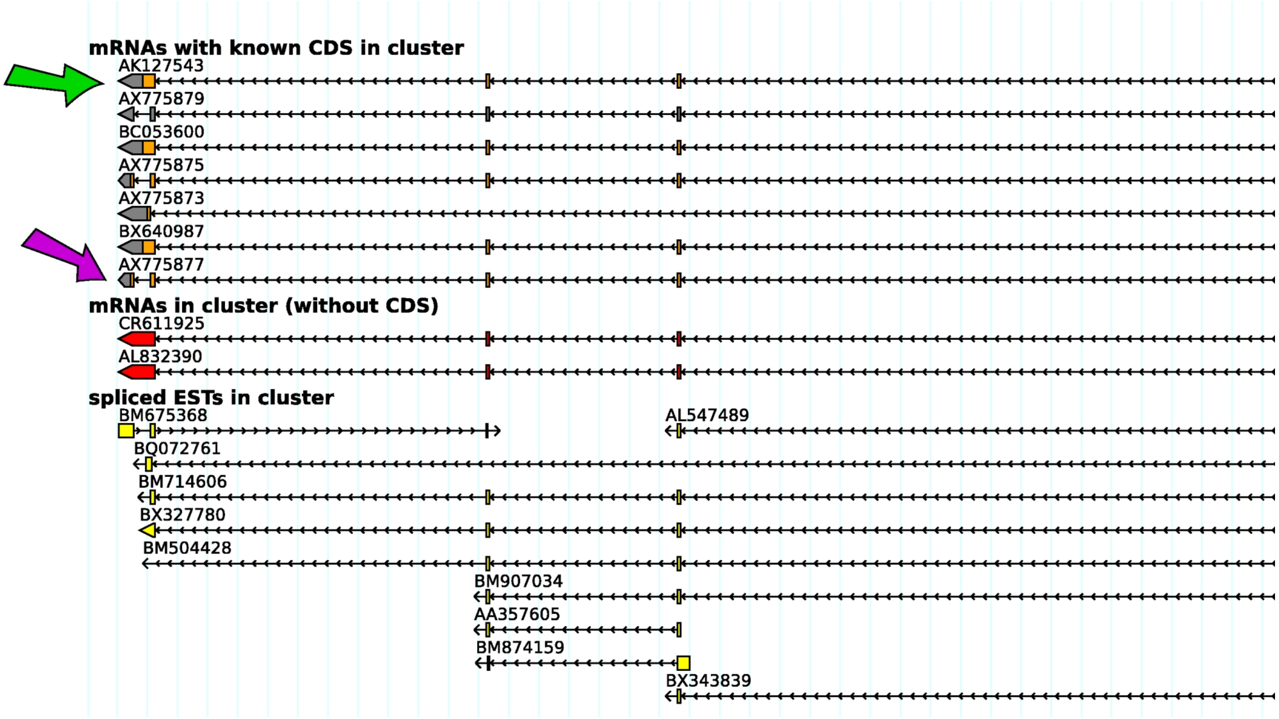

mRNAs with known CDS in cluster
AK127543
AX775879
BC053600
AX775875
AX775873
BX640987
AX775877
mRNAs in cluster (without CDS)
CR611925
AL832390
spliced ESTs in cluster
BM675368
AL547489
BQ072761
BM714606
BX327780
BM504428
BM907034
AA357605
BM874159
BX343839

(D)

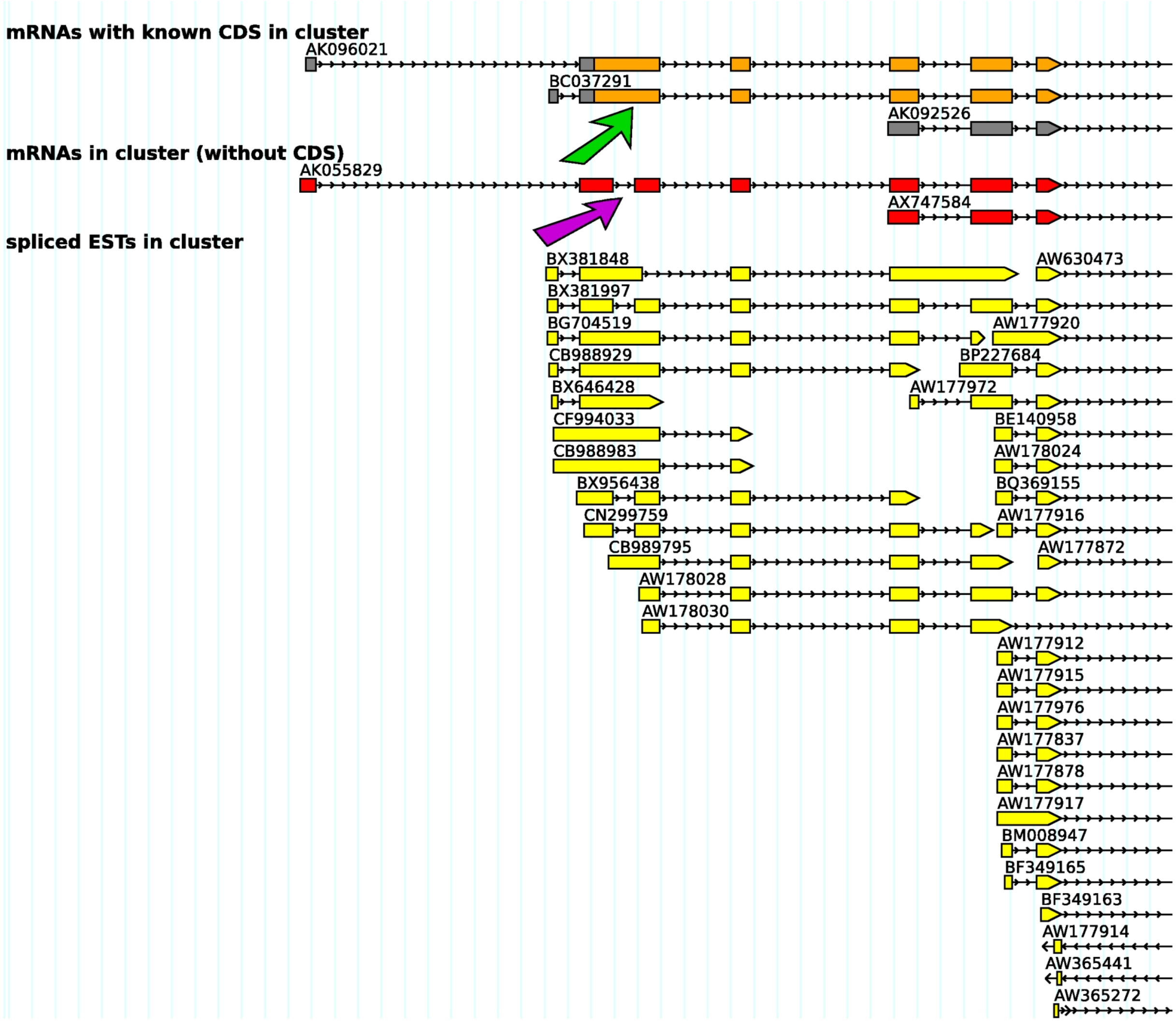

Figure S1. Examples of real IR events in different cDNA clusters. (A, B) Low-RIF events. (A) intron retention in the extremity of a cDNA (B) intron retention flanked by introns on both sides. (C, D) High-RIF events. Arrows point to intron retaining and intron defining cDNAs.
